# Burden Analysis of Rare Microdeletions Suggests a Strong Impact of Neurodevelopmental Genes in Genetic Generalised Epilepsies

**DOI:** 10.1371/journal.pgen.1005226

**Published:** 2015-05-07

**Authors:** Dennis Lal, Ann-Kathrin Ruppert, Holger Trucks, Herbert Schulz, Carolien G. de Kovel, Dorothée Kasteleijn-Nolst Trenité, Anja C. M. Sonsma, Bobby P. Koeleman, Dick Lindhout, Yvonne G. Weber, Holger Lerche, Claudia Kapser, Christoph J. Schankin, Wolfram S. Kunz, Rainer Surges, Christian E. Elger, Verena Gaus, Bettina Schmitz, Ingo Helbig, Hiltrud Muhle, Ulrich Stephani, Karl M. Klein, Felix Rosenow, Bernd A. Neubauer, Eva M. Reinthaler, Fritz Zimprich, Martha Feucht, Rikke S. Møller, Helle Hjalgrim, Peter De Jonghe, Arvid Suls, Wolfgang Lieb, Andre Franke, Konstantin Strauch, Christian Gieger, Claudia Schurmann, Ulf Schminke, Peter Nürnberg, EPICURE Consortium, Thomas Sander

**Affiliations:** 1 Cologne Center for Genomics (CCG), University of Cologne, Cologne, Germany; 2 Cologne Excellence Cluster on Cellular Stress Responses in Aging-Associated Diseases (CECAD), University of Cologne, Cologne, Germany; 3 Department of Neuropediatrics, University Medical Center Giessen and Marburg, Giessen, Germany; 4 EPICURE Consortium; 5 Department of Medical Genetics, University Medical Center Utrecht, Utrecht, The Netherlands; 6 SEIN Epilepsy Institute in the Netherlands, Hoofddorp, The Netherlands; 7 Department of Neurology and Epileptology, Hertie Institute of Clinical Brain Research, University of Tübingen, Tübingen, Germany; 8 Department of Neurology, University of Munich Hospital—Großhadern, Munich, Germany; 9 Department of Epileptology, University Clinics Bonn, Bonn, Germany; 10 Department of Neurology, Charité University Medicine, Campus Virchow Clinic, Berlin, Germany; 11 Department of Neurology, Vivantes Humboldt-Klinikum, Berlin, Germany; 12 Department of Neuropediatrics, University Medical Center Schleswig-Holstein (Kiel Campus), Kiel, Germany; 13 Epilepsy-Center Hessen, Department of Neurology, Philipps-University Marburg, Marburg, Germany; 14 Epilepsy Center Frankfurt Rhein-Main, Department of Neurology, Johann Wolfgang Goethe University Frankfurt, Frankfurt, Germany; 15 Department of Neuropediatrics, University Medical Center Giessen and Marburg, Giessen, Germany; 16 Department of Neurology, Medical University of Vienna, Vienna, Austria; 17 Department of Pediatrics and Neonatology, Medical University of Vienna, Vienna, Austria; 18 Department of Neurology, Danish Epilepsy Centre, Dianalund, Denmark; 19 Institute for Regional Health Services, University of Southern Denmark, Odense, Denmark; 20 Neurogenetics Group, VIB Department of Molecular Genetics, University of Antwerp, Antwerp, Belgium; 21 Laboratory of Neurogenetics, Institute Born-Bunge, University of Antwerp, Antwerp, Belgium; 22 Institute of Epidemiology and Biobank Popgen, Christian Albrechts University, Kiel, Germany; 23 Institute of Clinical Molecular Biology, Christian-Albrechts-University of Kiel, Kiel, Germany; 24 Institute of Genetic Epidemiology, Helmholtz Zentrum München—German Research Center for Environmental Health, Neuherberg, Germany; 25 Institute of Medical Informatics, Biometry and Epidemiology, and Chair of Genetic Epidemiology, Ludwig-Maximilians-University, Munich, Germany; 26 Research Unit of Molecular Epidemiology, Helmholtz Zentrum München—German Research Center for Environmental Health, Neuherberg, Germany; 27 Institute of Epidemiology II, Helmholtz Zentrum München—German Research Center for Environmental Health, Neuherberg, Germany; 28 Interfaculty Institute for Genetics and Functional Genomics, Ernst Moritz Arndt University, Greifswald, Germany; 29 Department of Neurology, University Medicine Greifswald, Ernst Moritz Arndt University, Greifswald, Germany; 30 Center for Molecular Medicine Cologne (CMMC), University of Cologne, Cologne, Germany; The University of North Carolina at Chapel Hill, UNITED STATES

## Abstract

Genetic generalised epilepsy (GGE) is the most common form of genetic epilepsy, accounting for 20% of all epilepsies. Genomic copy number variations (CNVs) constitute important genetic risk factors of common GGE syndromes. In our present genome-wide burden analysis, large (≥ 400 kb) and rare (< 1%) autosomal microdeletions with high calling confidence (≥ 200 markers) were assessed by the Affymetrix SNP 6.0 array in European case-control cohorts of 1,366 GGE patients and 5,234 ancestry-matched controls. We aimed to: 1) assess the microdeletion burden in common GGE syndromes, 2) estimate the relative contribution of recurrent microdeletions at genomic rearrangement hotspots and non-recurrent microdeletions, and 3) identify potential candidate genes for GGE. We found a significant excess of microdeletions in 7.3% of GGE patients compared to 4.0% in controls (*P* = 1.8 x 10^-7^; OR = 1.9). Recurrent microdeletions at seven known genomic hotspots accounted for 36.9% of all microdeletions identified in the GGE cohort and showed a 7.5-fold increased burden (*P* = 2.6 x 10^-17^) relative to controls. Microdeletions affecting either a gene previously implicated in neurodevelopmental disorders (*P* = 8.0 x 10^-18^, OR = 4.6) or an evolutionarily conserved brain-expressed gene related to autism spectrum disorder (*P* = 1.3 x 10^-12^, OR = 4.1) were significantly enriched in the GGE patients. Microdeletions found only in GGE patients harboured a high proportion of genes previously associated with epilepsy and neuropsychiatric disorders (*NRXN1*, *RBFOX1*, *PCDH7*, *KCNA2*, *EPM2A*, *RORB*, *PLCB1*). Our results demonstrate that the significantly increased burden of large and rare microdeletions in GGE patients is largely confined to recurrent hotspot microdeletions and microdeletions affecting neurodevelopmental genes, suggesting a strong impact of fundamental neurodevelopmental processes in the pathogenesis of common GGE syndromes.

## Author Summary

Epilepsy affects about 4% of the general population during lifetime. The genetic generalised epilepsies (GGEs) represent the most common group of epilepsies with predominant genetic aetiology, accounting for 20% of all epilepsies. Despite their strong heritability, the genetic basis of the majority of patients with GGE remains elusive. Genomic microdeletions constitute a significant source of genetic risk factors for epilepsies. The present genome-wide burden analysis in 1,366 European patients with GGE and 5,234 ancestry-matched controls explored the role of large and rare microdeletions (size ≥ 400 kb, frequency < 1%) in the complex genetic architecture of common GGE syndromes. Our results revealed a 2-fold excess of microdeletions in GGE patients relative to the population controls, 2) a 7-fold increased burden for known hotspot microdeletions (15q11.2, 15q13.3, 16p13.11, 22q11.2) previously associated with a wide range of neurodevelopmental disorders, and 3) a more than 4-fold enrichment of microdeletions carrying a gene implicated in neurodevelopmental disorders. Our findings reinforce emerging evidence that genes affected by microdeletions in GGE patients have a strong impact in fundamental neurodevelopmental processes and dissect novel candidate genes involved in epileptogenesis.

## Introduction

The epilepsies comprise a clinically heterogeneous group of neurological disorders defined by recurrent spontaneous seizures due to paroxysmal excessive and synchronous neuronal activity in the brain [1]. Epilepsy affects about 4% of the general population during their lifetime [2] and about 40% of all epilepsies are thought to have a strong genetic contribution. The genetic generalised epilepsies (GGEs) represent the most common group of epilepsies with predominant genetic aetiology, accounting for 20% of all epilepsies [3]. Their clinical features are characterised by unprovoked generalised seizures with age-related onset, generalised spike and wave discharges on the electroencephalogram and no evidence for an acquired cause [4,5]. Despite their strong familial aggregation and heritability [6–9], the genetic architecture of common GGE syndromes is likely to display a biological spectrum, in which a small fraction (1–2%) follows monogenic inheritance, whereas the majority of GGE patients presumably display an oligo-/polygenic predisposition with extensive genetic heterogeneity [10]. Although causative mutations for rare GGE with monogenic inheritance have been identified in genes primarily affecting neuronal excitability, synaptic transmission, and neurodevelopmental processes [11,12], the genetic basis of the majority of patients with GGE remains largely unsolved.

Genomic copy number variations (CNVs) constitute a significant source of genetic risk factors for common focal and generalised epilepsies [13–20]. By targeted screening of rearrangements at genomic hotspots associated with neurodevelopmental disorders [21], we previously identified recurrent microdeletions at 15q11.2, 15q13.3 and 16p13.11 as important genetic risk factors of common GGE syndromes [14,16,17,22–24]. The microdeletions at 15q13.3 and 16p13.11 represent the most prevalent genetic determinants of GGE identified so far [14,16]. In addition, we were able to show that non-hotspot exonic microdeletions in three brain-expressed genes encoding gephyrin (*GPHN*) [25], neurexin 1 (*NRXN1*) [26] and the RNA-binding protein FOX1 (*RBFOX1*) [27] confer susceptibility of GGE. Although the GGE-associated microdeletions identified to date are individually rare (<1%), they cumulatively account for a significant fraction of the genetic burden in more than 3% of patients with common GGE syndromes [14–16,22].

In the present genome-wide burden analysis, we used the Affymetrix SNP 6.0 array to screen large (≥ 400 kb) and rare (< 1%) autosomal microdeletions with high calling confidence (≥ 200 markers) in European case-control cohorts of 1,366 GGE patients and 5,234 population controls. We aimed to: 1) assess the genetic burden of large and rare microdeletions in common GGE syndromes, 2) evaluate the contribution of recurrent hotspot and unique microdeletions to the genetic burden of GGE, and 3) identify novel candidate genes for GGE. Specifically, we tested the hypothesis whether microdeletions affecting genes involved in neurodevelopmental processes account for a significant fraction of the genetic risk of GGE syndromes.

## Results

### Burden analysis of autosomal microdeletions

We identified 103 microdeletions in 100 out of 1,366 GGE patients compared to 214 microdeletions in 208 out of 5,234 controls (S1 Table). Overall, 7.3% of patients with GGE carried at least one microdeletion compared to 4.0% in controls (*P* = 1.77 x 10^–7^; OR = 1.91, 95%-CI: 1.48–2.46) (Table 1). We observed a marginal increase in microdeletion frequency in the GGE patients when we considered only microdeletions affecting either at least one protein-coding RefSeq gene (n = 18,299; *P* = 5.86 x 10^–7^; OR = 1.95, 95%-CI: 1.48–2.57) or at least one brain-expressed gene (n = 8,878; *P* = 1.38 x 10^–7^; OR = 2.19, 95%-CI: 1.61–2.98) (Table 1). Likewise, the median size of microdeletions was larger in the GGE patients (713 kb; interquartile range (IQR) = 523 kb—1,537 kb) compared to controls (589 kb; IQR = 488–930 kb; *P* = 3.99 x 10^–3^; Wilcoxon-Mann-Whitney-Test). The number of individuals carrying at least two microdeletions did not differ significantly between the GGE patients (n = 3) and controls (n = 6; *P* = 0.40, Fisher´s exact test). The microdeletion burden was similar for males (7.2%) and females (7.4%) affected by GGE (*P* = 0.91; OR = 0.97, 95%-CI: 0.63–1.52). The distribution of GGE subsyndromes did not differ between 100 GGE patients carrying a microdeletion (33 JME, 50 CAE/JAE, 17 EGTCS/EGMA) and the group of 1,266 GGE patients without a large and rare microdeletion (507 JME, 548 CAE/JAE, 211 EGTCS/EGMA; *P* > 0.15).

**Table 1:** Microdeletion burden analysis.

Microdeletions	Genes N	GGE N = 1,366	CTR N = 5,234	*P*-value^	OR, 95%-CI
**Autosomal microdeletions**		100	208	**1.77E-07**	1.91; 1.48–2.46
RefSeq NM genes	18,299	85	172	**5.86E-07**	1.95; 1.48–2.57
Brain-expressed genes	8,878	70	126	**1.38E-07**	2.19; 1.61–2.98
Hotspot microdeletions		38	20	**2.61E-17**	7.46; 4.20–13.3
**Gene-set enrichment**					
Neurodevelopmental genes^#^	1,547	59	51	**8.02E-18**	4.58; 3.09–6.82
ASD-related genes*	1,669	45	43	**1.29E-12**	4.11; 2.64–6.40
**Control gene-sets**					
Randomly selected genes	3,256	12	35	0.412	1.32; 0.65–2.64
Not brain-expressed genes	3,837	13	58	0.618	0.86; 0.45–1.62

GGE, genetic generalised epilepsy; CTR: population control; RefSeq NM genes: autosomal protein-coding NM annotated genes of the human reference sequence database, genome build GRCh37/hg19; Brain-expressed genes: autosomal brain-expressed genes specified by a log(reads per kb per million reads) > 3.32 of the BrainSpan RNA-Seq transcriptome dataset [28]; Hotspot microdeletions: recurrent microdeletions at genomic rearrangement hotspots [29]: Neurodevelopmental genes: autosomal genes associated with neurodevelopmental disorders based on literature and database queries [30]; Autism spectrum related genes: autosomal brain-expressed genes that were selectively enriched for deleterious exonic *de novo* mutations in ASD individuals relative to their healthy siblings [31]; Autosomal genes not expressed in the brain, defined by the BrainSpan RNA-Seq transcriptome database [28]; ^*P*-value: *P*-value obtained for χ2-test with df = 1; OR, 95%-CI, odds ratio with 95% confidence interval.

### GGE-related spectrum of microdeletions

The spectrum of 103 microdeletions identified in 100 GGE patients comprised: 1) 38 (36.9%) recurrent microdeletions at seven known genomic rearrangement hotspots previously associated with a wide range of neurodevelopmental disorders [29], 2) 27 (26.2%) genic microdeletions that were detected only in the GGE patients, 3) 16 (15.5%) microdeletions without a protein-coding RefSeq gene and that were not present in the controls, and 4) 22 (21.4%) non-hotspot microdeletions which overlap with the microdeletions identified in the controls (S1 Table). Most prominent was the 7.5-fold excess of recurrent hotspot microdeletions in the GGE patients compared to the controls (*P* = 2.61 x 10^–17^; OR = 7.46, 95%-CI: 4.20–13.33; χ^2^-test, df = 1) (Table 2). Overall, 2.8% (n = 38) of 1,366 GGE patients carried one of the known recurrent microdeletions at 1q21.1 (n = 1), 15q11.2 (n = 13), 15q13.3 (n = 11), 16p11.2 (n = 1), 16p12 (n = 3), 16p13.11 (n = 6) and 22q11.2 (n = 3), whereas these hotspot microdeletions were observed only in 0.4% (n = 20) of 5,234 population controls (S1 and S2 Figs). Significant associations with GGE patients were found for single hotspot microdeletions at 15q11.2 (*P* = 4.18 x 10^–4^; OR = 3.58; 95%-CI: 1.58–8.09, χ^2^-test, df = 1), 15q13.3 (*P* = 2.89 x 10^–8^, Fisher´s exact test), 16p13.11 (*P* = 1.48 x 10^–3^; OR = 11.48, 95%-CI: 2.05–116.5, Fisher´s exact test), and 22q11.2 (*P* = 8.85 x 10^–3^, Fisher´s exact test). All hotspot microdeletions in GGE patients identified by SNP arrays were validated by TaqMan qPCR. Altogether, the present findings highlight the cumulative impact of the recurrent microdeletions at 15q11.2, 15q13.3, 16p13.11 and 22q11.2 on the genetic risk of common GGE syndromes.

**Table 2:** Recurrent microdeletions at rearrangement hotspots.

Recurrent Microdeletion	hg19 position	GGE N = 1,366	CTR N = 5,234	Candidate Gene	*P*-value	OR, 95%-CI
1q21.1	chr1: 146.5–147.5	1	1	*GJA8*	0.37^**#**^	3.8; 0.05–300
15q11.2	chr15: 22.8–23.1	13	14	*CYFIP1*	4.18E-04*	3.5; 1.6–8.1
15q13.3	chr15: 31.3–32.5	11	0	*CHRNA7*	**2.89E-08** ^**#**^	Inf; 9.7-Inf
16p13.11	chr16: 15.0–16.3	6	2	*NDE1*	**1.48E-03** ^**#**^	11.5; 2.1–117
16p12	chr16:21.9–22.5	3	2		0.06^**#**^	5.8; 0.7–69
16p11.2	chr16: 29.6–30.2	1	1	*PRRT2*	0.37^**#**^	3.8; 0.05–300
22q11.2	chr22: 18.8–21.6	3	0	*SNAP29*	**8.85E-03** ^**#**^	Inf; 1.6-Inf
**Total**	** **	**38**	**20**	** **	2.61E-17*	**7.5; 4.20–13.3**

GGE, genetic generalised epilepsy; CTR: population control; *P*-value: *P*-value obtained for ^**#**^ Fisher´s exact test or * χ2-test with df = 1; OR, 95%-CI, odds ratio with 95% confidence interval.

Besides the recurrent hotspot microdeletions, we identified 27 GGE patients carrying a genic microdeletion that was not observed in the controls (Table 3 and S1 Table and S3 Fig). These microdeletions affected 158 protein-coding RefSeq genes and exhibited an enrichment of genes previously associated with epilepsy (*NRXN1*, *RBFOX1*, *PCDH7*, *KCNA2*, *EPM2A*, *RORB*, *PLCB1*) and neuropsychiatric disorders (*DPYD*, *CADM2*, *PARK2*, *GRM8*, *TSNARE1*, *TPH2*, *MACROD2*) (Table 3). Microdeletions involving *NRXN1* exons 1–2 were observed in two GGE patients with genetic absence epilepsies [26]. In addition, two partially overlapping microdeletions were identified in the chromosomal region 8q24.3 encompassing the genes encoding the t-SNARE domain containing 1 protein (*TSNARE1;* chr8: 143,293,441–143,484,601) and the brain-specific angiogenesis inhibitor 1 (*BAI1*; chr8: 143,545,376–143,626,368). All other unique microdeletions occurred only once. The microdeletions affecting the neuronal genes, *NRXN1* and *RBFOX1*, have been reported in two previous publications [26,27].

**Table 3:** Gene-disrupting microdeletions found only in patients with genetic generalised epilepsy.

Sample-ID	Syndrome	Chr	Start	End	Candidate gene	Disease/Trait	References
EC-CAE428	6/CAE,gsw	1	97005643	97712686	*DPYD*	SCZ,AUT	[32–34]
EC-EGTCS014	14/EGTCS,gsw	1	110606081	111393713	***KCNA2***,*ALX3*	EPI,ID	[35–39]
EC-CAE333^#^	5/CAE,gsw	2	50979977	51453231	***NRXN1***	GGE	[26,40,41]
EC-JAE085^#^	12/JAE,gsw	2	51080429	51682854	***NRXN1***	GGE	[26,40,41]
EC-JME399	13/JME,gsw	2	130275170	130762880	*RAB6C*		
EC-JME104	13/JME,gsw	3	85017098	85603757	*CADM2*	ADHD	[42,43]
EC-CAE040	7/CAE,gsw	3	165317672	166886252	*BCHE*	ADHD,SCZ	[44]
EC-JME445	26/JME	4	27778687	31233363	***PCDH7***	GGE,EPI	[45–48]
EC-CAE099	6/CAE,gsw	5	275875	1257621	*SLC6A19*,*TERT*		
EC-JME481	14/JME,gsw	5	28059042	31736582	*DROSHA*,*CDH6*	SCZ	[49–51]
EC-CAE347	9/CAE,gsw	6	144444363	146880409	***EPM2A***,*GRM1*	SCZ,EPI	[52,53]
EC-CAE204	8/CAE,gsw	6	162801345	163287279	*PARK2*		[54–58]
EC-JME461	4/CAE	7	124586130	126665734	*GRM8*	ASD,ADHD	[59–61]
EC-CAE158	8/CAE,gsw	7	143223069	143873940	*FAM11C5*,*FAM115A*		
EC-EGTCS130	25/EGTCS,gsw	8	99979097	100538070	*VPS13B*		
EC-JME417	17/JME,gsw	8	142563566	143798641	*TSNARE1*,*BAI1*,*ARC*	SCZ,BPD	[62]
EC-JAE119	16/JAE,gsw	8	142850077	143549806	*TSNARE1*,*BAI1*	SCZ,BPD	[62]
EC-CAE300	10/CAE	9	76601085	77182821	***RORB***	EPI,ID	[63–65]
EC-JME005	12/JME,gsw	10	27836576	28429513	*MPP7*,*ARMC4*,*MKX*	ID	[66]
EC-JME054	20/JME,gsw	11	4167416	5262622	*C11orf40*,*TRIM68*		
EC-JME425	13/JME	12	72135173	73995884	*TBC1D15*,*TPH2*	ADHD	[67–69]
EC-JME642	15JME,gsw	15	84915113	85726714	*WDR73*,*PDE8A*	MCP	[70]
EC-CAE286*	3/CAE,gsw	16	5615773	6512138	***RBFOX1***	ASD,SCZ,EPI	[27,71–73]
EC-CAE226	6/CAE,gsw	18	13982898	14969710	*ZNF519*		
EC-CAE161	7/CAE,gsw	20	8099277	8572225	***PLCB1***	EE,EPI,SCZ	[54,74–77]
EC-EGTCS119	15/EGTCS,gsw	20	14902412	15312347	*MACROD2*	AUT,ADHD	[78–82]
EC-JME101	24/JME	21	45866974	48096945	***ADARB1***,*S100B*		[83,84]

GGE, genetic generalised epilepsy; CTR: population control; Chr: chromosome, start/end: genomic start and end point of the deleted segment, hg19; ^*P*-value: type-1 error rate for a χ2-test with df = 1; OR, 95%-CI, odds ratio with 95% confidence interval. Disease phenotype: ASD: autism spectrum disorder, ADHD: attention deficit hyperactivity disorder, AN: anorexia nervosa, AUT: autism, BPD: bipolar disorder, EE: epileptic encephalopathy, EPI: epilepsy, ID: intellectual disability, MCP: microcephaly, SCZ: schizophrenia; GGE syndromes: CAE: childhood absence epilepsy, JAE: juvenile absence epilepsy, JME: juvenile myoclonic epilepsy, EGMA: epilepsy with generalised tonic-clonic seizures alone predominantly on awakening, EGTCS: epilepsy with generalised tonic-clonic seizures alone, gsw: generalised spike and wave discharges on the electroencephalogram, number/: age-at-onset of afebrile generalised seizures. # previously published in [26] and * [27]. Bold gene symbols indicate genes previously implicated in epileptogenesis.

### Gene-set enrichment analyses of neurodevelopmental genes

To explore the hypothesis whether neurodevelopmental genes affected by the microdeletions have an impact on the genetic risk of common GGE syndromes, we performed enrichment analyses of the deleted genes, using two previously published sets of genes implicated in neurodevelopmental disorders (ND): 1) ND-related genes (n = 1,547) compiled by literature and database queries [30], and 2) genes implicated in autism spectrum disorder (ASD-related genes) comprising 1,669 brain-expressed genes with an enrichment of deleterious exonic *de novo* mutations in ASD [31]. Microdeletions carrying at least one ND-related gene were 4.6-fold enriched in the GGE patients as compared to the controls (*P* = 8.02 x 10^–18^; OR = 4.58, 95%-CI: 3.09–6.82) (Table 1). Likewise, microdeletions encompassing at least one ASD-related gene showed a 4.1-fold excess in the GGE patients relative to the controls (*P* = 1.29 x 10^–12^; OR = 4.11, 95%-CI: 2.64–6.40) (Table 1). To explore the impact of neurodevelopmental genes that are not covered by the recurrent hotspot microdeletions, we combined the ND- and ASD-related gene lists [30,31] and removed all genes affected by observed recurrent hotspot microdeletions. Non-recurrent microdeletions carrying at least one of the 2,495 selected ND/ASD-related genes showed a 2.3-fold excess in GGE patients (n = 1,328) compared to control subjects (n = 5,214), when individuals with recurrent hotspot microdeletions were excluded (*P* = 4.56 x 10^–4^; OR = 2.48, 95%-CI: 1.42–4.30). To rule out an artificial enrichment of microdeletions in the GGE patients, we compiled two control gene assemblies comprising: 1) 3,256 randomly selected autosomal protein-coding RefSeq genes, and 2) 3,837 autosomal protein-coding RefSeq genes not expressed in the brain [28]. Both control gene assemblies did not show evidence for an increase of the microdeletion burden in GGE patients compared to controls (*P* > 0.40) (Table 1).

### Functional enrichment and network analyses

The Disease Association Protein-Protein Link Evaluator (DAPPLE v2.0) tool [85] was applied to identify significant physical connectivity among proteins encoded by genes affected by microdeletions. Therefore, we separately tested the gene assemblies for the GGE patients and the control subjects. Based on an initial regional query we extracted 191 seed genes from 103 microdeletions found in the GGE patients and 221 seed genes from 214 microdeletions observed in controls. There was an overlap of 61 genes between the two assemblies. DAPPLE network analyses revealed a significant enrichment for direct connections between the seed genes (*P* = 0.01) in the GGE microdeletion carriers, while the control gene network did not show evidence for an enrichment (*P* = 0.40). Finally, in GGE we found eleven genes with significant connectivity: *PLCB1* (*P* = 0.002), *GRM1* (*P* = 0.002), *ARC* (*P* = 0.002), *CNTN6* (*P* = 0.015), *CHL1* (*P* = 0.033), *BAI1* (*P* = 0.033), *CYFIP1* (*P* = 0.040), *TRIP13* (*P* = 0.042), *MAPK3* (*P* = 0.044), *GJ8* (*P* = 0.048), and *KCNA2* (*P* = 0.050) (S4 Fig).

Utilising the Enrichr tool [86], functional enrichment analysis of the gene assembly affected by the microdeletions in the GGE patients revealed a significant enrichment of the MGI Mammalian Phenotype term "abnormal emotion/affect behaviour" (MP:0002572; *P*_adj_ = 1.30 x 10^–3^) and the GO biological process term “cognition” (GO:0050890; *P*_adj_ = 0.012) (Table 4). Enrichr network analysis identified one significant PPI Hub in the GGE patients based on an enrichment of nine deleted genes (*ARC*, *TJP1*, *MAPK3*, *MYH11*, *EXOC3*, *NRXN1*, *PARK2*, *PLCB1*, *GRM1*) among 219 network genes (*P*_adj_ = 0.018), for which *GRIN2B* encodes the shared interacting protein.

**Table 4:** Functional gene enrichment and network analysis.

Gene-set library	*P*_*adj*_-value	Genes
***MGI Mammalian Phenotype***		
abnormal emotion/affect behaviour (MP:0002572)	1.30E-03	*ARC NTAN1 RORB PARK2 GRM1 GRM8 APBA2 CHRNA7 CHL1 TPH2 COMT/ZDHHC/RTN4R*
***GO biological process***		
cognition (GO:0050890)	0.012	*ARC OR52B4 EPM2A NTAN1 PARK2 NRXN1 S100B BCHE CYFIP1 CHRNA7 PLCB1 CHL1 DGCR2/COMT*
***PPI Hub Proteins***		
GRIN2B	0.018	*ARC TJP1 MAPK3 MYH11 EXOC3 NRXN1 PARK2 PLCB1 GRM1*

Significant gene-set enrichments on 329 genes deleted in GGE patients revealed an enrichment of GRIN2B interacting proteins, genes of the MGI abnormal emotion/affect behaviour annotation and of the GO cognition annotation. Segmental clusters of genes belonging to a gene family were removed. Positional clustering of genes physically linked on a microdeletion is indicated by a slash between the gene symbols.

## Discussion

### High burden driven by recurrent hotspot microdeletions

The present burden analysis applied a screening strategy that focused on both large (≥ 400 kb, ≥ 200 markers) and rare (< 1%) autosomal microdeletions to ensure a high calling accuracy [87] and to enrich pathogenic microdeletions among confounding benign copy number polymorphisms [88–90]. We found a significant 1.9-fold excess of microdeletions in the patients with GGE compared to the controls (Table 1). Overall, 7.3% of the 1,366 GGE patients carried at least one microdeletion compared to 4.0% in 5,234 controls. These findings highlight the important impact of microdeletions on the genetic susceptibility of common GGE syndromes with an attributable risk of about 3.3%.

The spectrum of 103 microdeletions identified in the GGE patients contained a high proportion (36.9%) of recurrent microdeletions at genomic rearrangement hotspots, known to play a pathogenic role in a wide range of neuropsychiatric disorders including epilepsy [13,91,92]. In total, 2.8% of the GGE patients carried one of the known pathogenic hotspot microdeletions at 1q21.1, 15q11.2, 15q13.3, 16p11.2, 16p12, 16p13.11 and 22q11.2 (Table 2), whereas these hotspot microdeletions were found only in 0.4% of the population controls (S1 and S2 Figs). Although these hotspot microdeletions are individually rare (< 1%), they collectively result in a 7.5-fold increased burden in the GGE patients and a population-attributable risk of about 2.4%. A previous genome-wide CNV search in epilepsies observed a similar cumulative prevalence of recurrent hotspot microdeletions in 3.5% out of 399 GGE patients [16]. Likewise, a targeted screening of the microdeletions at 15q11.2, 15q13.3 and 16p13.11 showed a cumulative frequency of 3.1% in 359 GGE patients and an even higher frequency of 10% in 60 GGE patients with intellectual disability [17]. Several other CNV studies targeting these genomic rearrangement hotspots also emphasised a substantial impact of recurrent microdeletions at 15q11.2, 15q13.3 and 16p13.11 in the pathogenesis of GGE and other epilepsies [14–20,22–24,93,94]. To our knowledge, this is the first study demonstrating a significant association of the recurrent microdeletion at 22q11.2 with GGE. Re-evaluation of the clinical records of three GGE patients carrying a 22q11.2 microdeletion revealed additional congenital and developmental features fitting to known conditions of the 22q11.2 deletion syndrome (OMIN 188400/192430). GGE patient (EC-EGMA094) had a moderate psychomotoric retardation, patient (EC-EGTCS145) was affected by a cleft palate and an atrial septal defect, and patient (EC-EGTCS044) had a mild impairment of his motoric coordination during childhood, moderate learning disabilities and hypocalcaemia, highlighting the 22q11.2 deletion syndrome as a multisystem disorder with high penetrance and variable phenotypic spectrum [95]. According to our ascertainment scheme [96], the present GGE patients with recurrent microdeletions did not exhibit severe intellectual disability or severe psychiatric comorbidities at the age of exploration but may evolve psychiatric disorders at later age. Considering the published CNV studies of epilepsies [14–20,24], meta-analyses may demonstrate an association of the less frequent recurrent hotspot microdeletions at 16p11.2 and 16p12 with GGE. Haploinsufficiency of *CYFIP1* at 15q11.2 [97], *CHRNA7* at 15q13.3 [98], *NDE1* at 16p13.11 [99] and *PRRT2* at 16p11.2 [100] has been implicated as risk-conferring mechanism for epilepsy and other neurodevelopmental phenotypes [88,89,91].

Functional-enrichment, pathway and network analyses showed significant connectivity of genes affected by microdeletions in GGE patients (S4 Fig) and a significant enrichment for the MGI Molecular Function category "abnormal emotion/affect behaviour" (MP:0002572) as well as the GO biological process term “cognition” (GO:0050890). The protein-protein interaction analyses highlight several genes that have been implicated in epileptogenesis (*CYFIP1*, *GRIN2B*, *KCNA2*, *NRXN1*, *PLCB1*) [14,16,26,39,74,75,97] and neurodevelopmental processes (*ARC*, *GRM1*, *PARK2*) [51,52,55,57–59].

### Enrichment of microdeletions involving neurodevelopmental genes

In line with our neurodevelopmental hypothesis, we found a significant 4.6-fold excess of microdeletions carrying at least one ND-related gene [30] and a 4.1-fold enrichment of microdeletions affecting at least one ASD-related gene [31] in the GGE patients compared to the control subjects. In contrast, the two control gene assemblies did not show an increase of the microdeletion burden in GGE patients compared to controls (*P* > 0.40). Accordingly, the intriguing enrichment of ND- and ASD-related genes demonstrates that genes involved in neurodevelopmental processes play an important role in the epileptogenesis of common GGE syndromes. Notably, the moderate overlap of the previously published assemblies of ND- and ASD-related genes implicates a large number of neurodevelopmental genes contributing to the risk of common GGE syndromes and extensive genetic heterogeneity. The emerging overlap of gene-disrupting microdeletions and the rapidly evolving landscape of loss-of-function gene mutations in rare and common epilepsy syndromes will facilitate the prioritisation of causal epilepsy genes and the elucidation of the leading molecular pathways of epileptogenesis [101,102].

### Non-hotspot microdeletions implicating potential GGE genes

We identified 27 gene-covering microdeletions in non-hotspot genomic regions that were present only in GGE patients (Table 3 and S3 Fig). These autosomal microdeletions involved several genes previously implicated in epilepsy and neurodevelopmental disorders. Although it remains challenging to distinguish benign and pathogenic microdeletions, several of these contain plausible candidate genes for epilepsy. Of particular interest were seven genes at seven microdeletion loci that have been associated with epilepsy.

Three of the epilepsy-associated microdeletions have been reported in two previous publications demonstrating an association of microdeletions affecting the 5´-terminal exons of the neuronal genes encoding the adhesion molecule neurexin 1 (*NRXN1*; 2p16.3, chr2: 50,145,642–51,259,673, hg19) and the splicing regulator RNA-binding protein fox-1 homolog (*RBFOX1*; 16p13.3, chr16: 5,289,468–7,763,341, hg19) [26,27]. The microdeletions involving *NRXN1* exons 1–2 were observed in two female GGE patients with genetic absence epilepsies [26]. The 5´-terminal untranslated *RBFOX1* exons 1–2 were deleted in a female patient with childhood absence epilepsy [27]. Deleterious mutations and microdeletions of the genes, *NRXN1* and *RBFOX1*, have been reported in a large number of patients with a broad range of neuropsychiatric disorders, who were frequently also affected by epilepsy [40,41,54,72,81]. A recent study demonstrated that the splicing regulator *Rbfox1* controls neuronal excitation in the mammalian brain and the *Rbfox1* knockout in mice results in an increased susceptibility to spontaneous and kainic acid-induced seizures [71]. Furthermore, molecular, cellular, and clinical evidence supports a pivotal role of *RBFOX1* in human neurodevelopmental disorders [73,103].

A 3.45 Mb microdeletion harbouring the protocadherin *PCDH7* gene (chromosomal location: 4p15.1, chr4: 30,721,950–31,148,422, hg19) was found in a female GGE subject with juvenile myoclonic epilepsy. An international GWAS meta-analysis including 8,696 epilepsy patients and 26,157 controls highlights *PCDH7* as susceptibility gene for epilepsy in general and GGE syndromes in particular [45]. The *PCHD7* gene encodes a calcium-dependent adhesion protein that is expressed in neurons of thalamocortical circuits and the hippocampus [46]. *PCDH7* has been implicated as neuronal target gene of *MECP2* [47], the gene for Rett syndrome (OMIM #312750), which manifests as a progressive neurodevelopmental disorder with recurrent seizures. Moreover, mutations in the X-chromosomal protocadherin gene *PCDH19* cause epilepsy and intellectual disability in females [48]. These lines of evidence suggest an involvement of *PCDH7* in epileptogenesis.

A 788 kb microdeletion involving the Shaker-like voltage-gated potassium channel gene *KCNA2* (1p13, chr1: 111,136,002–111,174,096, hg19) was identified in a male GGE patient with generalised tonic-clonic seizures starting at the age of 14. The Kv1 subfamily plays an essential role in the initiation and shaping of action potentials, influencing action potential firing patterns and controlling neuronal excitability as well as seizure susceptibility [36,38,39]. De novo loss- or gain-of-function mutations in *KCNA2* have been identified to cause human epileptic encephalopathy [39]. Furthermore, the *Kcna2* knockout mice exhibit spontaneous seizures and have a reduced life span [35,37].

One female GGE patient with childhood absence epilepsy carried a 2.4 Mb microdeletion in the chromosomal region 6q24.6 encompassing two neuronally expressed genes encoding the metabotropic glutamate receptor type 1 (*GRM1*; chr6: 146,348,917–146,758,734, NM_001278065, hg19) and laforin (*EPM2A*; chr6: 145,946,439–146,056,991, NM_005670, hg19). Deleterious mutations in the *GRM1* gene have been found in patients with schizophrenia [52]. Also, familial segregation analysis of deleterious non-synonymous sequence variants revealed a co-segregation with multiple neuropsychiatric conditions including epilepsy in some families. Recessive mutations/microdeletions of *EPM2A* cause progressive myoclonic epilepsy type 2A (Lafora disease, OMIM #254780) [53].

A 582 kb microdeletion encompassing exon 1 of the gene encoding the RAR-related orphan receptor B (*RORB*; 9q21.13, chr9: 77,112,251–77,303,533, NM_006914, hg19) was found in a male patient with childhood absence epilepsy, overlapping with the critical region of a novel microdeletion syndrome at 9q21.13 characterised by intellectual disability, speech delay, facial dysmorphisms and epilepsy [63]. The *RORB* gene is a strong candidate for the neurological phenotype because *RORB* was deleted in all affected individuals [63], it is expressed in the cerebral cortex and thalamus, and genetic associations of *RORB* with bipolar disorder [64] and verbal intelligence [65] have been reported.

The gene encoding the enzyme phospholipase C-beta 1 (*PLCB1*; 20p12.3, chr20: 8,112,911–8,865,546, hg19) was partially deleted (exons 1–3, NM_015192, hg19) in a male GGE patient with childhood absence epilepsy. PLCB1 catalyses the generation of inositol 1,4,5-trisphoshate and diacylglycerol from phosphatidylinositol 4,5-bisphosphate, a key step in the intracellular transduction of many extracellular signals. Homozygous microdeletions of chromosome 20p12.3, disrupting the promoter region and first three coding exons of *PLCB1*, have previously been reported in two consanguineous families with early infantile epileptic encephalopathy [74]. Mutation analysis of a family with severe intractable epilepsy and neurodevelopmental delay revealed compound heterozygous mutations in *PLCB1* composed of a 476 kb microdeletion encompassing *PLCB1* and a deleterious *PLCB1* splice site mutation [75]. Girirajan et al. [54] found an enrichment of microdeletions and duplications involving the *PLCB1* gene in individuals with autism. Together, these findings implicate that the *PLCB1* gene contributes to the genetic risk of neurodevelopmental disorders including epilepsy.

In addition to the epilepsy-associated microdeletions, nine deleted genes have been previously implicated as genetic risk factors in a broad range of neuropsychiatric disorders. Unique hemizygous microdeletions in GGE patients involved *DPYD*/1p13.3 [32–34], *CADM2*/3p12.1 [43], *BCHE*/3q26.1 [44], *PARK2*/6q24 [54,55,57,58], *GRM8*/7q31.33 [59–61]. *TSNARE1*/8q24.3 [62], *MPP7-ARMC4-MKX*/10p12.1 [66], *TPH2*/12q21.1 [67–69], *MACROD2*/20p12.1 [78–81], and *ADARB1*/21q22.3 [83,84]. Notably, overlapping microdeletions encompassing *TSNARE1* at chromosome 8q24.3 in two GGE patients indicate its potential role in epileptogenesis. A recent GWAS meta-analysis of psychiatric disorders identified *TSNARE1* as susceptibility gene for schizophrenia, schizoaffective and bipolar disorders [62]. While the function of TSNARE1 remains elusive, bioinformatic predictions suggest a vertebrate-specific function in synaptic vesicle exocytosis [104]. Further studies will be necessary to disentangle the pathogenic genes and to elucidate their molecular pathways in neurodevelopmental disorders and epileptogenesis.

### Summary

Our burden analysis of large and rare autosomal microdeletions (size ≥ 400 kb, frequency < 1%) revealed: 1) a nearly 2-fold excess of microdeletions in GGE patients relative to the population controls, 2) a 7-fold increased burden for known hotspot microdeletions previously associated with neurodevelopmental disorders, and 3) a more than 4-fold enrichment of microdeletions carrying a gene implicated in neurodevelopmental disorders. Recurrent microdeletions at seven genomic rearrangement hotspots accounted for 37% of all microdeletions identified in the GGE patients and predominantly contributed to the excess of microdeletions in GGE patients. Comorbidity of GGE with other neurodevelopmental disorders, such as intellectual disability, ASD and schizophrenia, may result in even higher prevalence of recurrent hotspot microdeletions [17] and emphasises a valuable diagnostic contribution to the clinical management of these severely affected comorbid patients with GGE. The remarkable phenotypic variability observed for the recurrent hotspot microdeletions suggests a shared susceptibility of a wide range of neuropsychiatric disorders and GGE [105]. Several genes affected by microdeletions that were found only in GGE patients highlight novel candidate genes for GGE. Altogether, the present findings reinforce converging lines of evidence that genes affected by microdeletions in GGE patients reside in fundamental neurodevelopmental processes.

## Materials and Methods

### Case-control cohorts

The study protocol was approved by the local institutional review boards of the contributing clinical centres. All study participants provided written informed consent. Genomic DNA samples of all study participants were processed by the Affymetrix SNP 6.0 array. For the genome-wide CNV burden analysis, we did not include individuals with excessive CNV counts (> 50 autosomal deletions per individual for deletions spanning > 40 kb in size and covering > 20 markers). In addition, we excluded all Affymetrix SNP 6.0 array data derived from lymphoblastoid cell lines because of the clonal source of the DNA which is prone to CNV artefacts compared to genomic DNA samples derived from blood cells [21]. All study participants were of self-reported North-Western European origin.

Unrelated GGE patients of European descent were ascertained through the primary diagnosis of a common GGE syndrome according to the classification of the International League Against Epilepsy [1,4]. The standardised protocols for phenotyping of GGE syndromes as well as inclusion and exclusion criteria are available online at: http://portal.ccg.uni-koeln.de/ccg/research/epilepsy-genetics/sampling-procedure/ [96]. GGE patients with a history of severe major psychiatric disorders (autism spectrum disorder, schizophrenia, affective disorder: recurrent episodes requiring pharmacotherapy or treatment in a hospital), or severe intellectual disability (no basic education, permanently requiring professional support in their daily life) were excluded. The GGE cohort comprised 1,366 patients (853 females, 513 males) with the following age-related GGE syndromes: childhood absence epilepsy (CAE, n = 398), juvenile absence epilepsy (JAE, n = 191), unspecified genetic absence epilepsy (GAE, n = 9), juvenile myoclonic epilepsy (JME, n = 540), epilepsies with generalised tonic-clonic seizures (GTCS) alone predominantly on awakening (EGMA, n = 94), and epilepsies with recurrent unprovoked GTCS alone starting before the age of 26 (EGTCS, n = 134). These 1,366 GGE patients were collected from Austria (n = 142), Belgium (n = 39), Denmark (n = 97), Germany (*n* = 801) and the Netherlands (n = 287). Notably, 1,052 of the GGE patients and 3,022 population controls investigated in the present study were part of a previous study that investigated six target microdeletions at genomic rearrangement hotspots [14].

Affymetrix SNP 6.0 data from 5,234 German population controls (2,559 females, 2,675 males) were obtained from three epidemiologically based cohorts: 1) KORA cohort from South Germany (n = 1,507) [106], 2) PopGen cohort from North Germany (*n* = 1,143) [107], and 3) SHIP cohort from East Germany (n = 2,584) [108]. The population controls were unscreened for epilepsy or major neuropsychiatric disorders. EIGENSTRAT principal component analysis [109] was applied to remove ancestry outliers and to match for European ancestry of the case-control cohorts [96].

### CNV analysis and screening of autosomal microdeletions

Genomic DNA samples were investigated by the Affymetrix Genome-Wide Human SNP Array 6.0 (Affymetrix, Santa Clara, CA, USA). CNV analysis was performed as previously described [14,22], using the Birdsuit algorithm implemented in the Affymetrix Genotyping Console version 4.1.1. All annotations refer to the genome build GRCh37/hg19. The present genome-wide burden analysis focused on rare and large autosomal microdeletions to ensure a high reliability of the microdeletion calls [87] and to enrich pathogenic microdeletions [88–90]. Therefore, we filtered out autosomal microdeletions with high calling confidence according to the following criteria: a) size ≥ 400 kb, b) coverage of ≥ 200 probe sets, and c) microdeletion frequency < 1% in the entire study sample. The microdeletion size of at least 400 kb was selected because all known pathogenic hotspot microdeletions identified in neurodevelopmental disorders exceed this size in CNV scans with the Affymetrix SNP Array 6.0 [29,88–90]. We did not include microduplications in the present burden analysis because the accuracy of CNV detection is lower for microduplications compared to microdeletions [110]. In particular, genomic DNA samples with substantial degradation are prone to spurious microduplication calls. Moreover, microduplications seem to exert pathogenic effects less frequently compared to microdeletions [88]. We excluded microdeletions with an overlap of > 10% with 12 chromosomal regions prone to artificial CNV calls according to a recently published "artefact list" [111]. For all QC-filtered microdeletions identified by SNP array screening, the segmental log2 ratios of the signal intensities and the SNP heterozygosity state were visually inspected by the Chromosome Analysis Suite v1.2.2 (Affymetrix, Santa Clara, CA, USA) to exclude spurious microdeletion calls. Validation of all 38 recurrent hotspot microdeletions and four GGE-associated microdeletions identified by SNP arrays in the GGE patients was carried out by real-time quantitative PCR (qPCR) according to the manufacturer´s instructions (Life Technologies, Carlsbad, CA, USA).

### Burden analyses

Overall burden analyses were carried out for three assemblies of autosomal microdeletions: 1) any microdeletion, 2) genic microdeletions encompassing at least one protein-coding RefSeq gene, defined by the largest NM gene transcript (n = 18,299, hg19), and 3) microdeletions affecting a brain-expressed gene (n = 8,878), specified by a log(RPKM) > 3.32 of the BrainSpan RNA-Seq transcriptome dataset (http://www.brainspan.org/) [28].

Specifically, we tested the hypothesis whether microdeletions affecting genes involved in neurodevelopmental processes account for a significant fraction of genetic risk of GGE syndromes. Therefore, we investigated two recently published assemblies of genes associated with neurodevelopmental disorders (ND): 1) ND-related genes compiling 1,547 genes that were associated with neuropsychiatric disorders, autism candidate genes and genes of known genomic disorders based on literature and database queries [30], and 2) ASD-related genes comprising 1,669 brain-expressed genes that were selectively enriched for deleterious exonic *de novo* mutations in ASD individuals relative to their healthy siblings [31]. To evaluate a spurious enrichment of microdeletions in the GGE patients relative to the population controls, we tested two control gene assemblies comprising: 1) 3,256 randomly selected autosomal genes, and 2) 3,837 autosomal genes not expressed in the brain [28], defined by the BrainSpan RNA-Seq transcriptome dataset. ND- and ASD-related genes, genes located in genomic rearrangement hotspots, or the artefact list were removed from the compiled control gene assemblies.

### Functional enrichment and network analyses

Functional-enrichment tests, pathway and network analyses were performed with the Disease Association Protein–Protein Link Evaluator version 2.0 program (DAPPLE v2.0; http://www.broadinstitute.org/mpg/dapple/dappleTMP.php; [85]) and the gene-set enrichment tool Enrichr (http://amp.pharm.mssm.edu/Enrichr/index.html; [86]). Therefore, we compiled two lists of genes affected by microdeletions in either the GGE patients (number of genes; n = 329; n = 191 regional seed genes) or the controls (n = 428 genes; n = 221 regional seed genes). There was an overlap of 103 genes (n = 61 seed genes) in both gene lists. To explore potential physical interactions among proteins encoded by deleted genes, DAPPLE uses experimentally validated, protein-protein interaction (PPI) databases to identify network and protein connectivity. Empirically, 1,000 random networks were generated by permutation to determine whether the connectivity of each seed protein with the PPI reference network was greater than that expected by chance.

The gene-set enrichment tool Enrichr was applied separately to explore patient and control lists of genes affected by microdeletions for an overlap with pathway gene-set libraries, specifically the database PPI Hub Proteins [112], and gene-set libraries created from Gene Ontology [113] as well as MGI Mammalian Phenotype terms [114]. A pathway or ontology term was considered as significantly enriched if the false discovery rate (FDR, Benjamini-Hochberg) was lower than 5% for an assembly of more than two genes and occurred only in the GGE patients but not in the controls.

### Statistical analyses

Burden analysis was performed by comparisons of the frequency of autosomal microdeletions in GGE patients and controls. The *P*-values and corresponding odds ratios (ORs) with the 95%-confidence intervals were calculated with a two-sided χ^2^-test or Fisher´s exact test if appropriate. The Wilcoxon-Mann-Whitney-Test was applied to compare differences in the genomic size of microdeletions. In addition, the individual burden of microdeletions was assessed for comparisons of microdeletion size. Nominal two-sided *P*-values < 0.05 were considered significant.

## Supporting Information

S1 Table Clinical information of microdeletion carriers and details on microdeletion calling and its genomic organisation. GGE, genetic generalised epilepsy, CTR, population control; Chr: chromosome, start/end: genomic start and end position of the microdeletion, hg19; GGE syndromes: CAE: childhood absence epilepsy, JAE: juvenile absence epilepsy, JME: juvenile myoclonic epilepsy, EGMA: epilepsy with generalised tonic-clonic seizures alone predominantly on awakening, EGTCS: epilepsy with generalised tonic-clonic seizures alone, gsw: generalised spike and wave discharges on the electroencephalogram; the number in front of the GGE syndromes refers to the individual age-at-onset of afebrile generalised seizures. Bold gene symbols indicate genes previously implicated in epileptogenesis. Previously published microdeletion: * [14], ** [26], *** [27].
(XLS)

S1 Fig Relative distribution of rare and large microdeletions in patients with genetic generalised epilepsies and controls. Microdeletions identified in patients with genetic generalised epilepsies (GGEs) and ethnically-matched European population controls differentiated by microdeletion type. Top left: Proportion of recurrent hotspot vs. non-recurrent microdeletions in population controls. Top right: Proportion of recurrent vs. non-recurrent deletions in GGE patients. Below: Relative distribution of recurrent microdeletions at seven genomic rearrangement hotspots in GGE patients.
(PDF)

S2 Fig Genomic organisation of recurrent microdeletions at seven genomic rearrangement hotspots in patients with genetic generalised epilepsies and population controls. Genomic organisation of recurrent microdeletions at the genomic rearrangement hotspots 1q21.1, 15q11.2, 15q13.3, 16p11.2, 16p12.2, 16p13.11 and 22q11.2. Tracks in red = patients with genetic generalised epilepsies (GGEs); tracks in beige = population controls. The annotations of genes (GRCh37/hg19) shown below are generated by the University of California, Santa Cruz Genome Browser (http://www.genome.ucsc.edu).
(PDF)

S3 Fig Gene-disrupting microdeletions found only in patients with genetic generalised epilepsies. Chr: chromosome, start/end: genomic start and end position of the deleted segment, hg19; GGE, genetic generalised epilepsy; GGE syndromes: CAE: childhood absence epilepsy, JAE: juvenile absence epilepsy, JME: juvenile myoclonic epilepsy, EGMA: epilepsy with generalised tonic-clonic seizures alone predominantly on awakening, EGTCS: epilepsy with generalised tonic-clonic seizures alone, gsw: generalised spike and wave discharges on the electroencephalogram, number/: age-at-onset of afebrile generalised seizures. Bold gene symbols indicate genes previously implicated in epileptogenesis. Signal intensity plots of microdeletions were visualized using the Affymetrix Chromosomal Analyze Suite software. Top track: Red bars represent the computed area of the observed microdeletions. Second track: Signal intensities (Log2 ratios) of a SNP or CN probe are represented by dots, one dot per probe. A segmental decline of consecutive probe signal intensities indicates a genomic deletion. Third track: Allele difference plot, a dot shift to zero indicates loss of heterozygosity which augments high-confidence deletion calling. Bottom track: Survey of the genomic organisation of the microdeletion generated with the University of California, Santa Cruz (UCSC) Genome Browser (http://www.genome.ucsc.edu). Genomic organisation of the microdeletion region: Red bars represent microdeletion size and genomic location in reference to genes affected by the microdeletion (generated with the UCSC Genome Browser, http://www.genome.ucsc.edu).
(PDF)

S4 Fig Protein-protein interaction networks analysis of genes affected by large and rare microdeletions in patients with genetic generalised epilepsies. DAPPLE direct networks derived from genes deleted in GGE patients. Depicted are the most connected networks in GGE. Connectivity is coloured from yellow to red describing low to high connection evidence respectively. Significant interactors are marked bold.
(PDF)
