# Supplementary material for: Burden Analysis of Rare Microdeletions Suggests a Strong Impact of Neurodevelopmental Genes in Genetic Generalised Epilepsies
Source: PLoS Genet. 2015 May 7;11(5):e1005226. doi: 10.1371/journal.pgen.1005226 (PMC4423931; doi:10.1371/journal.pgen.1005226)
Supplement: S1 Fig — Microdeletions identified in patients with genetic generalised epilepsies (GGEs) and ethnically-matched European population controls differentiated by microdeletion type. Top left: Proportion of recurrent hotspot vs. non-recurrent microdeletions in population controls. Top right: Proportion of recurrent vs. non-recurrent deletions in GGE patients. Below: Relative distribution of recurrent microdeletions at seven genomic rearrangement hotspots in GGE patients. (PDF) [file pgen.1005226.s002.pdf]

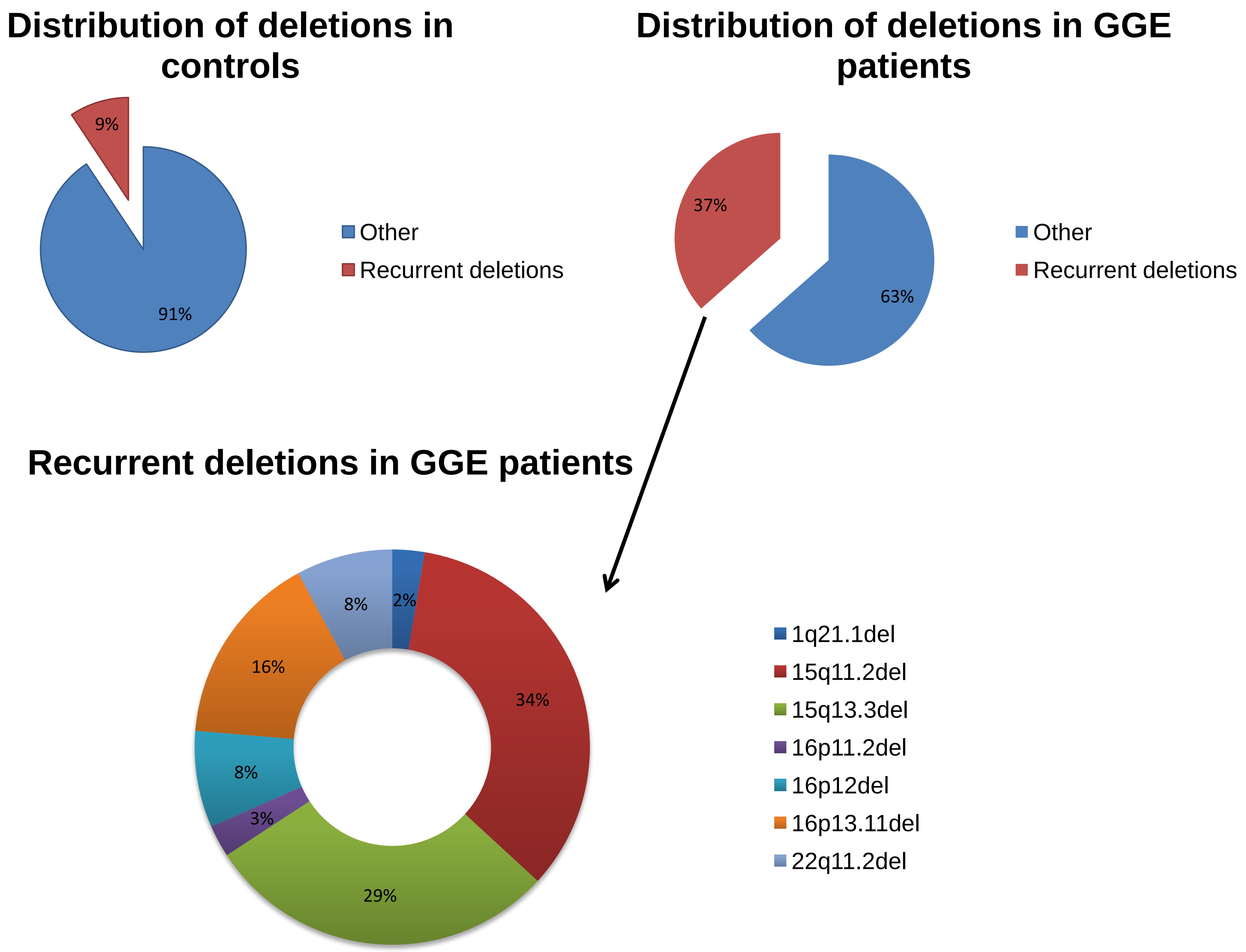
Distribution of deletions in controls
9%
91%
Other
Recurrent deletions
Distribution of deletions in GGE patients
37%
63%
Other
Recurrent deletions
Recurrent deletions in GGE patients
8%
2%
16%
34%
8%
3%
29%
1q21.1del
15q11.2del
15q13.3del
16p11.2del
16p12del
16p13.11del
22q11.2del
