# Supplementary material for: Burden Analysis of Rare Microdeletions Suggests a Strong Impact of Neurodevelopmental Genes in Genetic Generalised Epilepsies
Source: PLoS Genet. 2015 May 7;11(5):e1005226. doi: 10.1371/journal.pgen.1005226 (PMC4423931; doi:10.1371/journal.pgen.1005226)

**S2 Fig. Genomic organisation of recurrent microdeletions at seven genomic rearrangement hotspots in patients with genetic generalised epilepsies and population controls**

## Microdeletions at 1q21.1

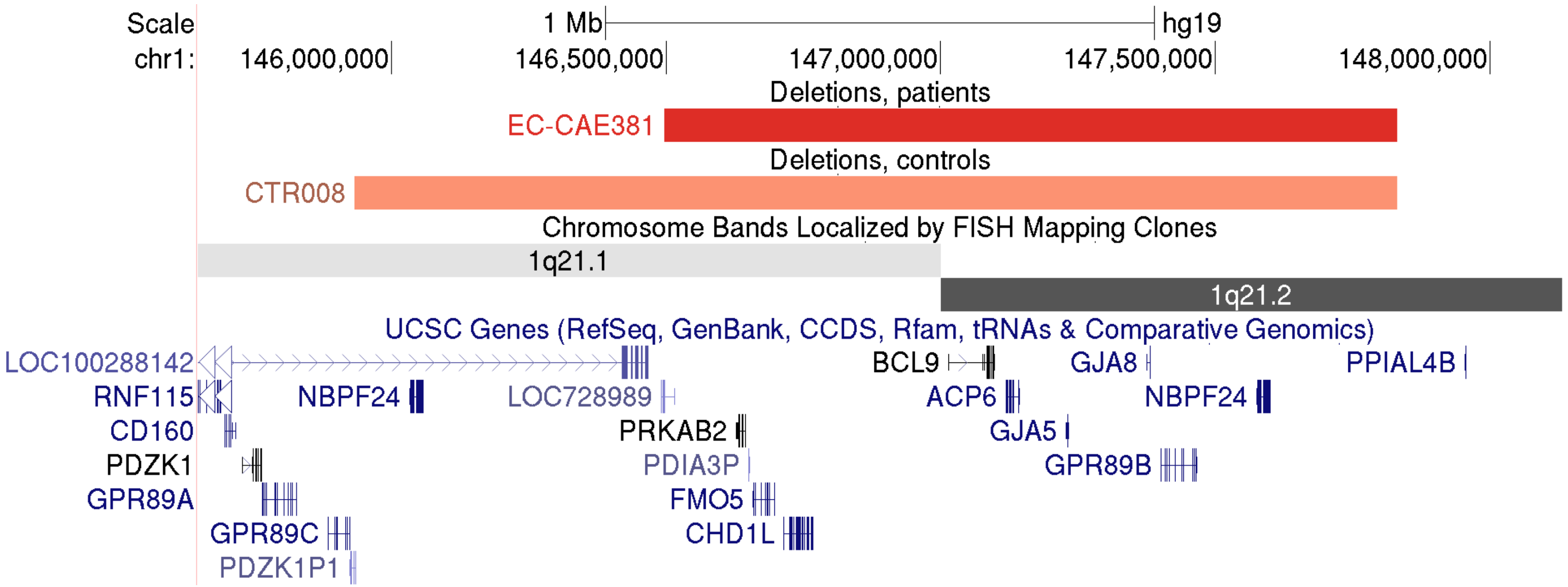

# Microdeletions at 15q11.2

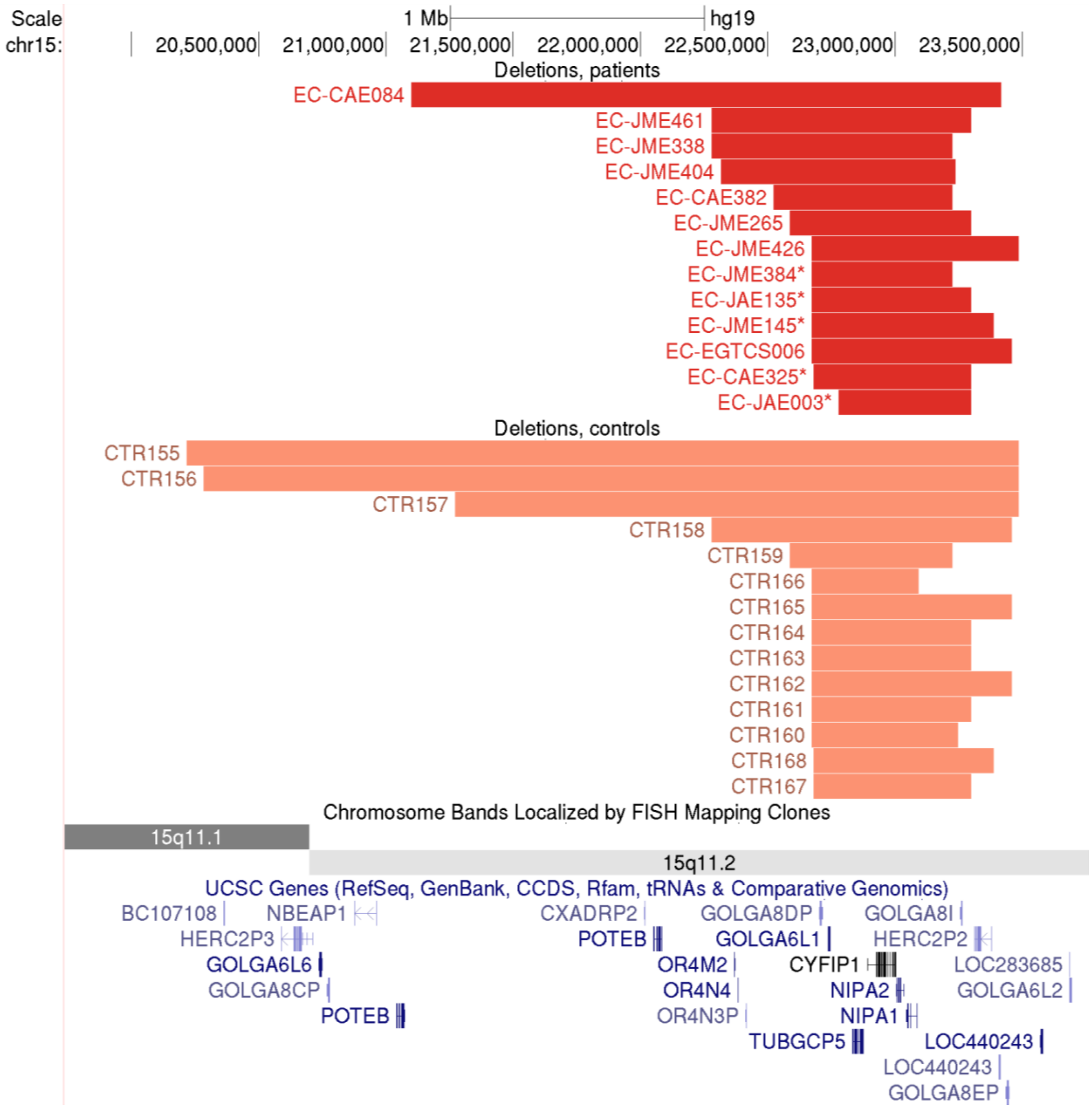

## Microdeletions at 15q13.3

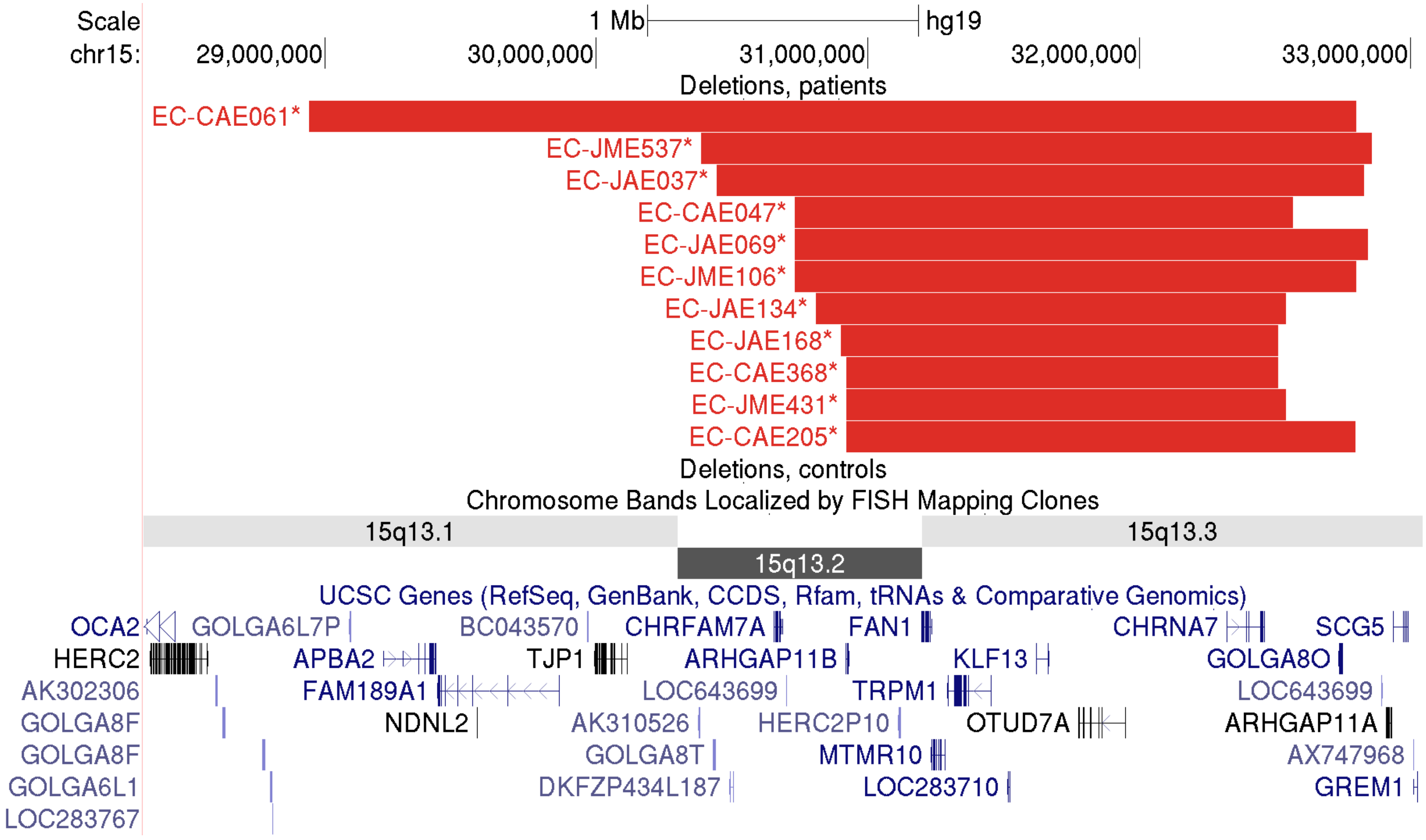

# Microdeletions at 16p11.2

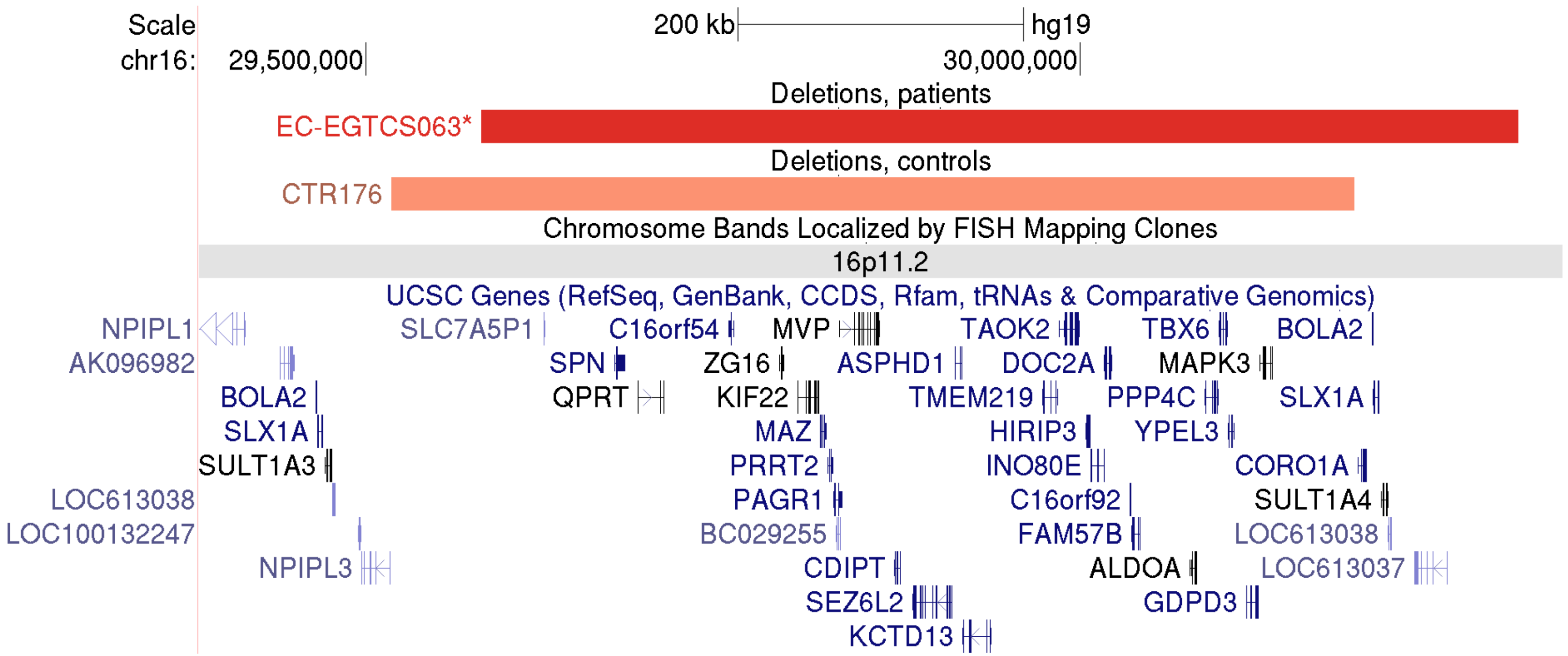

**Microdeletions at 16p12.2**

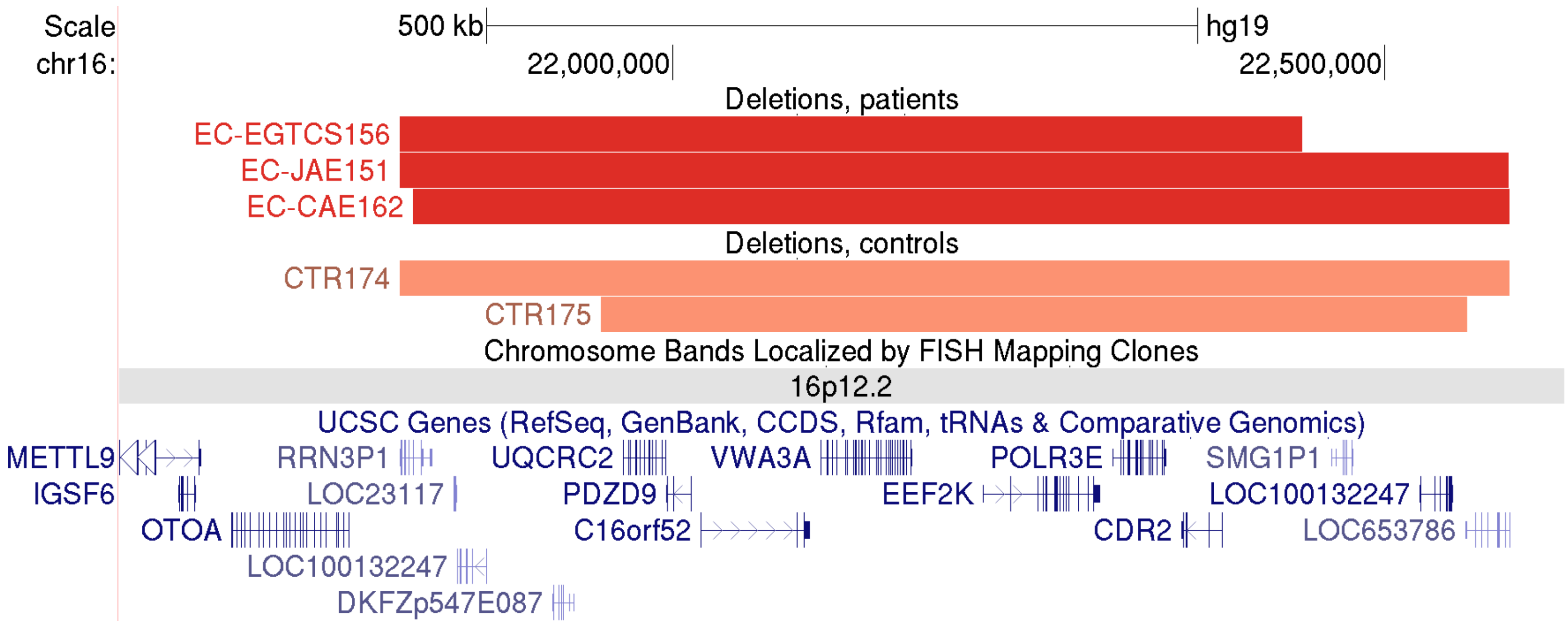

# Microdeletions at 16p13.3

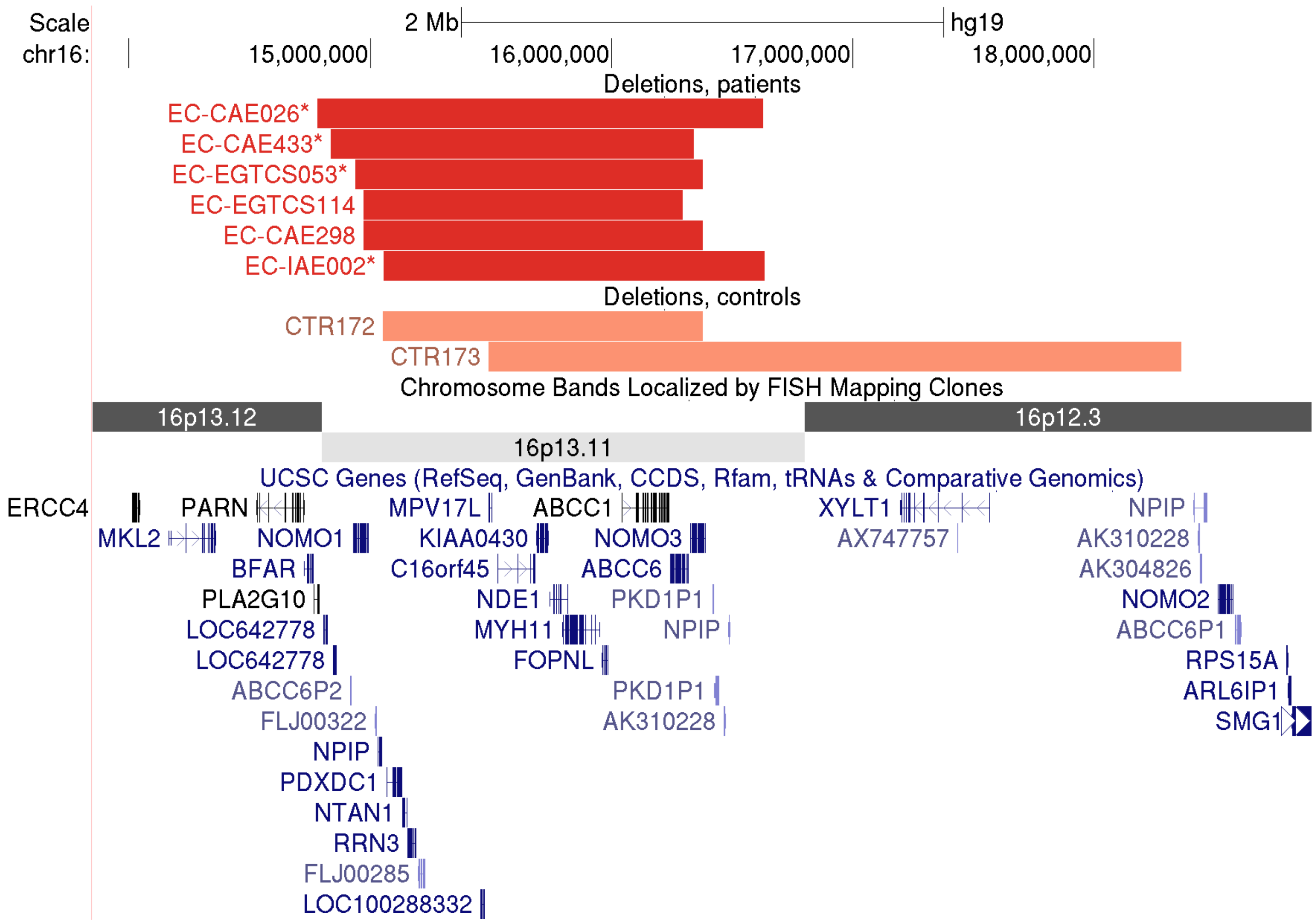

**Microdeletions at 22q11.2**

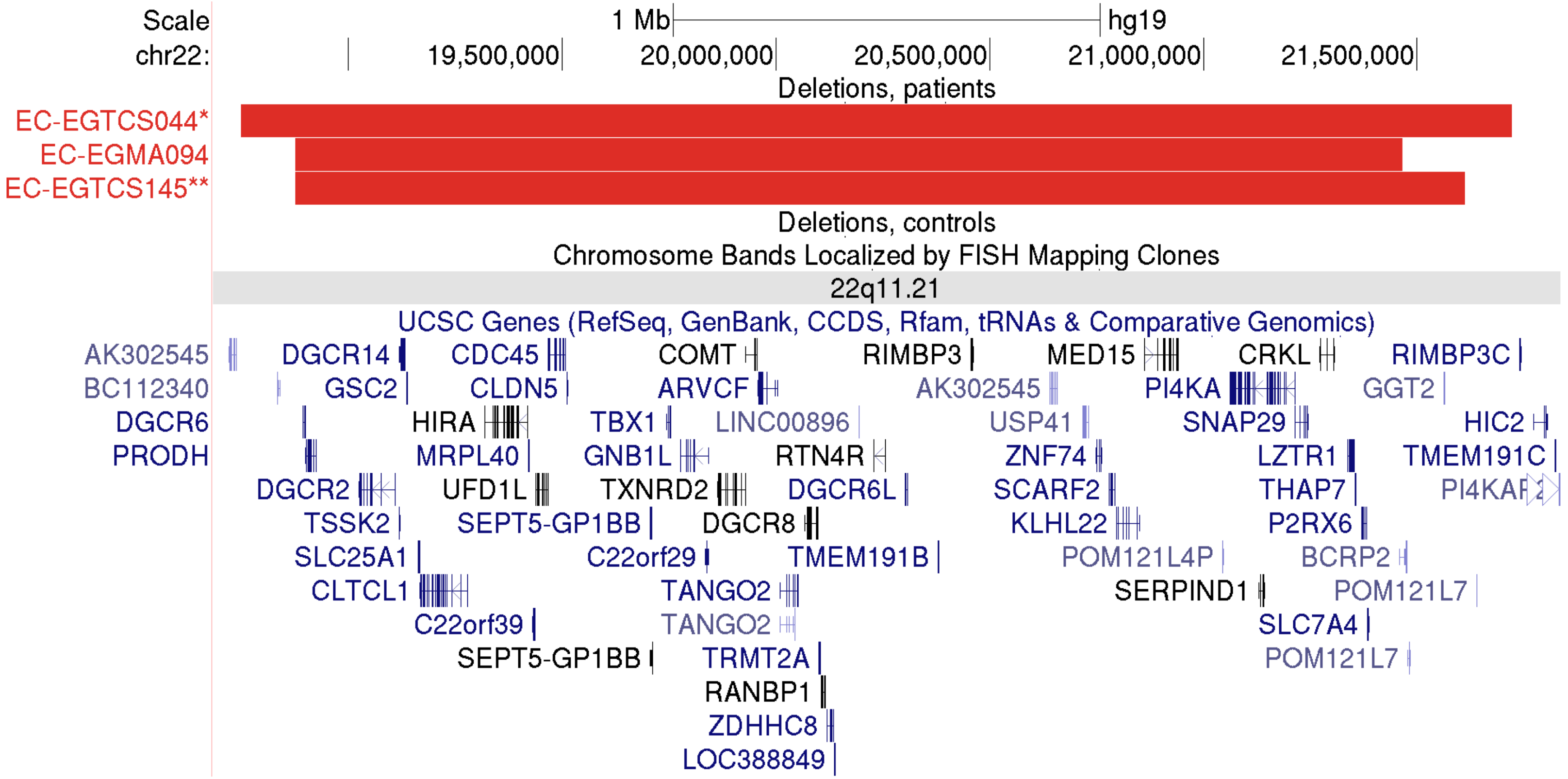

Supplement: S2 Fig — Genomic organisation of recurrent microdeletions at the genomic rearrangement hotspots 1q21.1, 15q11.2, 15q13.3, 16p11.2, 16p12.2, 16p13.11 and 22q11.2. Tracks in red = patients with genetic generalised epilepsies (GGEs); tracks in beige = population controls. The annotations of genes (GRCh37/hg19) shown below are generated by the University of California, Santa Cruz Genome Browser (http://www.genome.ucsc.edu). (PDF) [file pgen.1005226.s003.pdf]
