# Supplementary material for: Burden Analysis of Rare Microdeletions Suggests a Strong Impact of Neurodevelopmental Genes in Genetic Generalised Epilepsies
Source: PLoS Genet. 2015 May 7;11(5):e1005226. doi: 10.1371/journal.pgen.1005226 (PMC4423931; doi:10.1371/journal.pgen.1005226)

Sample-ID	GGE Syndrome	Chr	Start	End	Candidate gene
EC-CAE428	6/CAE,gsw	1	97005643	97712686	*DPYD*

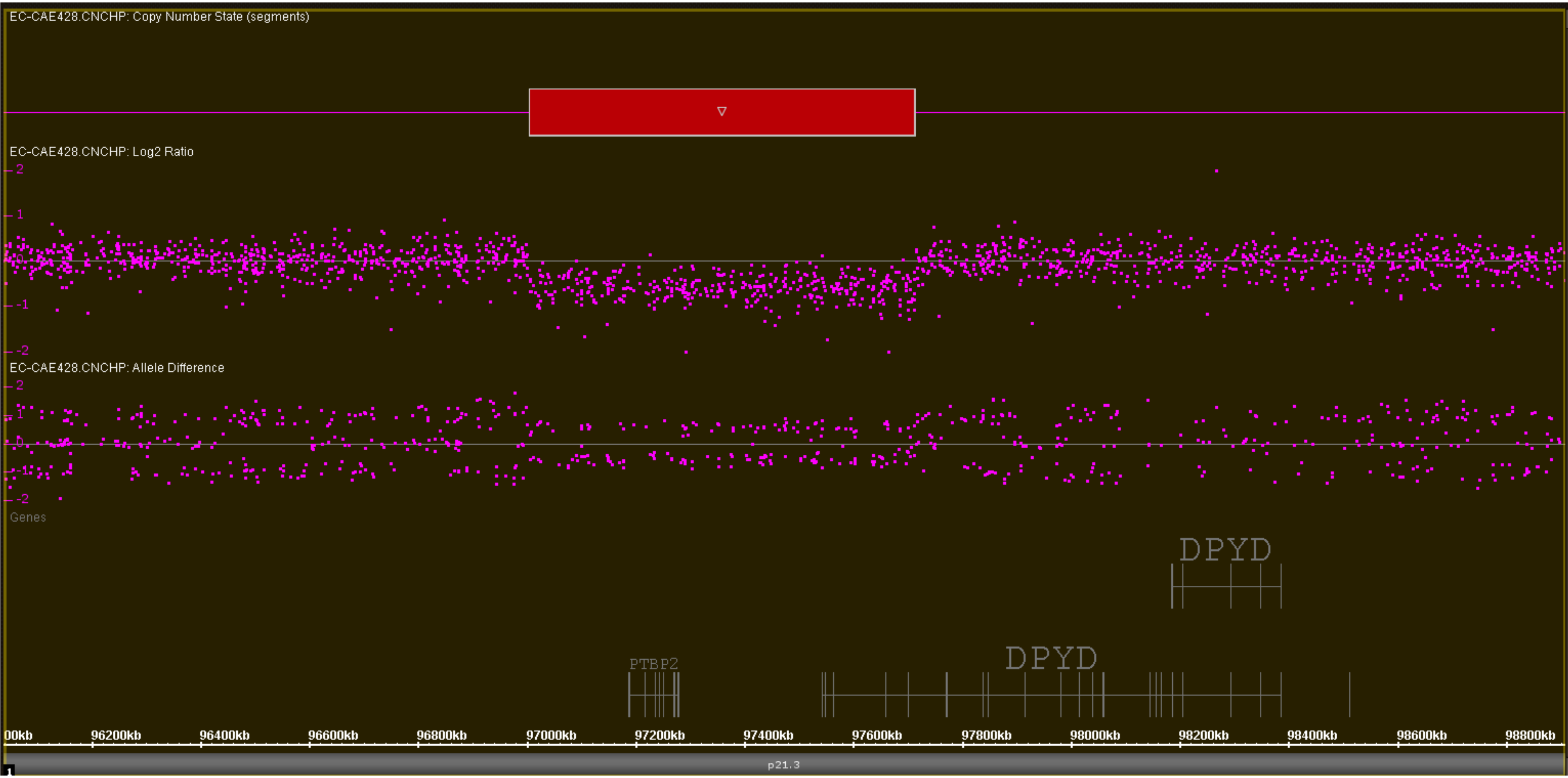

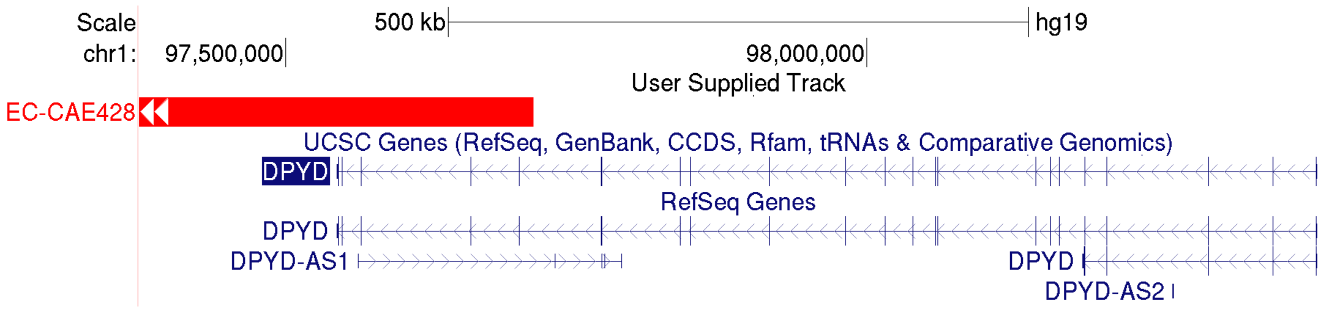

Sample-ID	GGE Syndrome	Chr	Start	End	Candidate gene
EC-EGTCS014	14/EGTCS,gsw	1	110606081	111393713	***KCNA2***,*ALX3*,*RBM15*

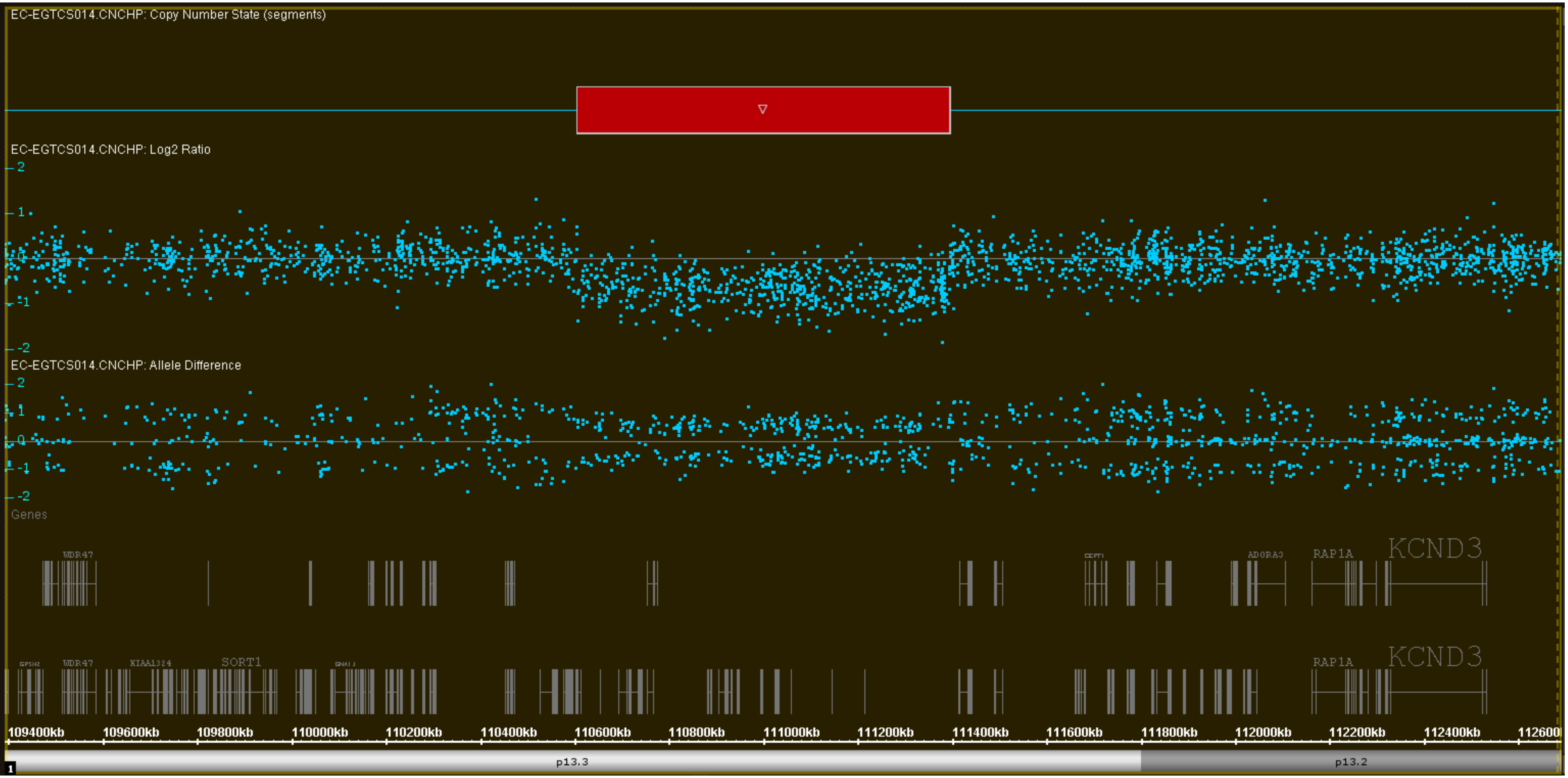

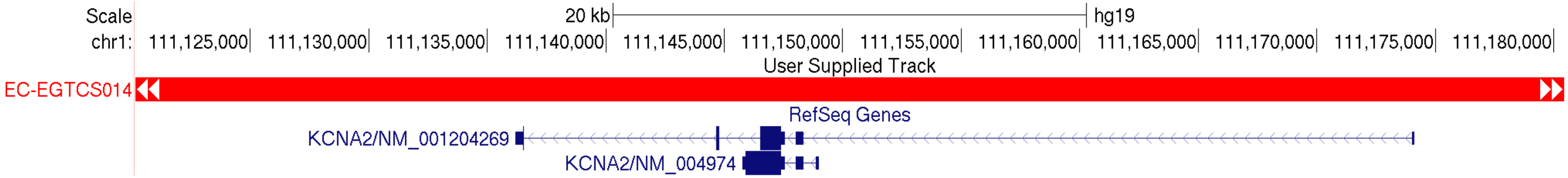

Sample-ID	GGE Syndrome	Chr	Start	End	Candidate gene
EC-CAE333	5/CAE,gsw	2	50979977	51453231	***NRXN1***

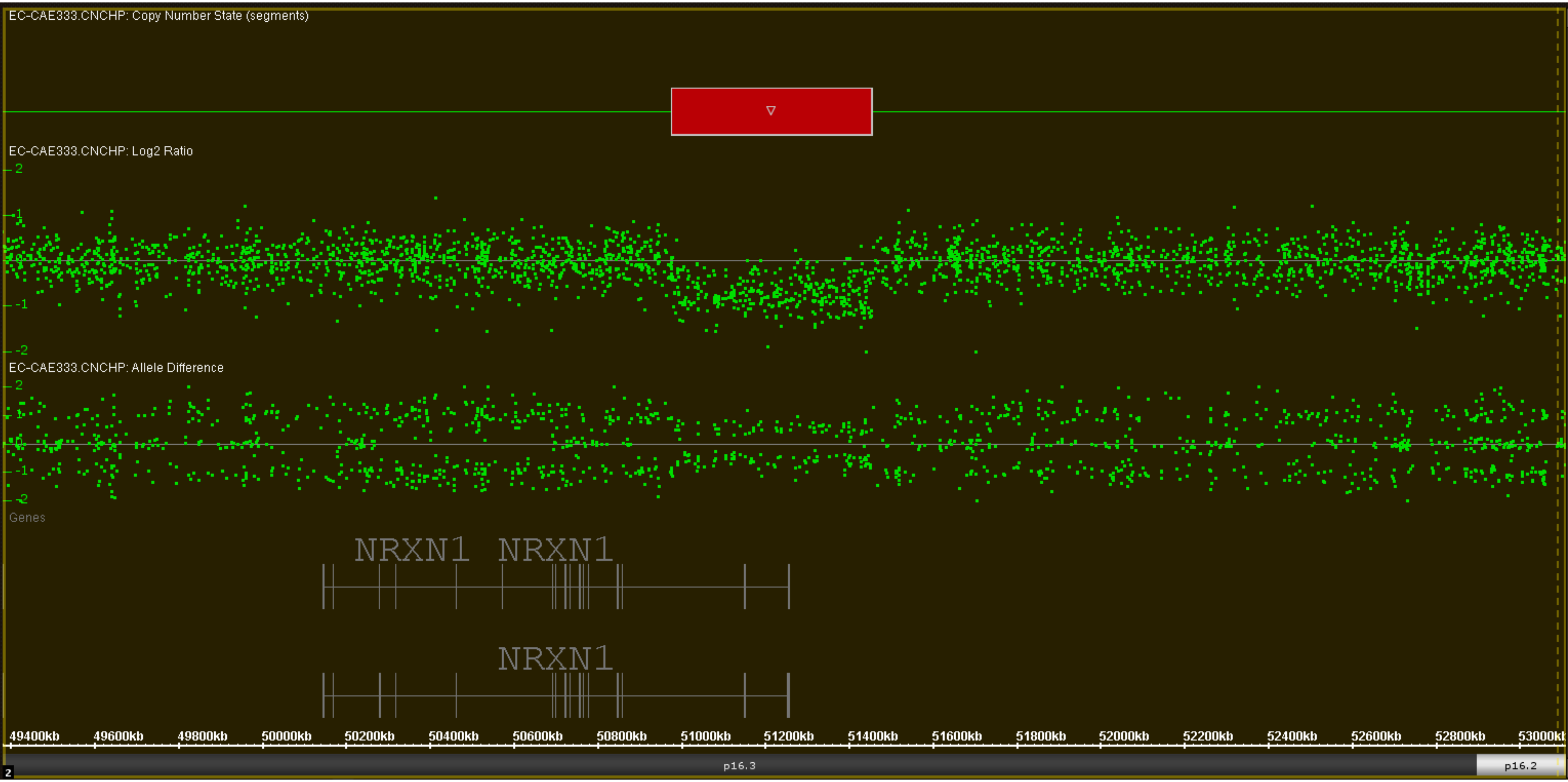

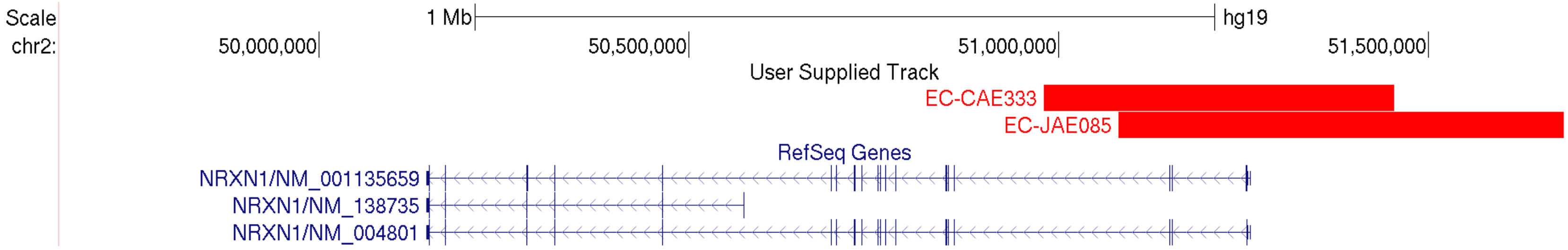

Sample-ID	GGE Syndrome	Chr	Start	End	Candidate gene
EC-JAE085	12/JAE,gsw	2	51080429	51682854	***NRXN1***

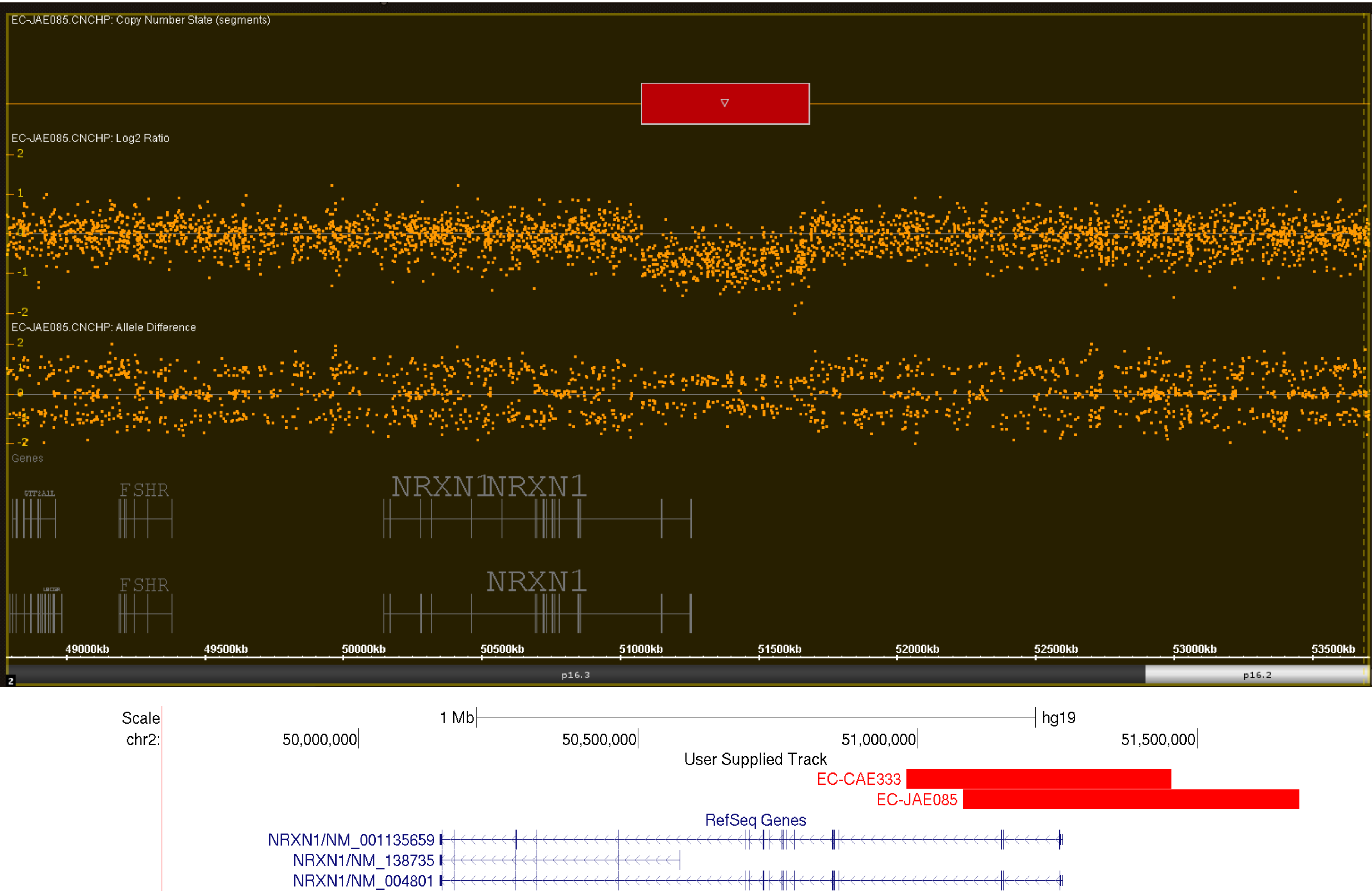

Sample-ID	GGE Syndrome	Chr	Start	End	Candidate gene
EC-JME399	13/JME,gsw	2	130275170	130762880	*RAB6C*

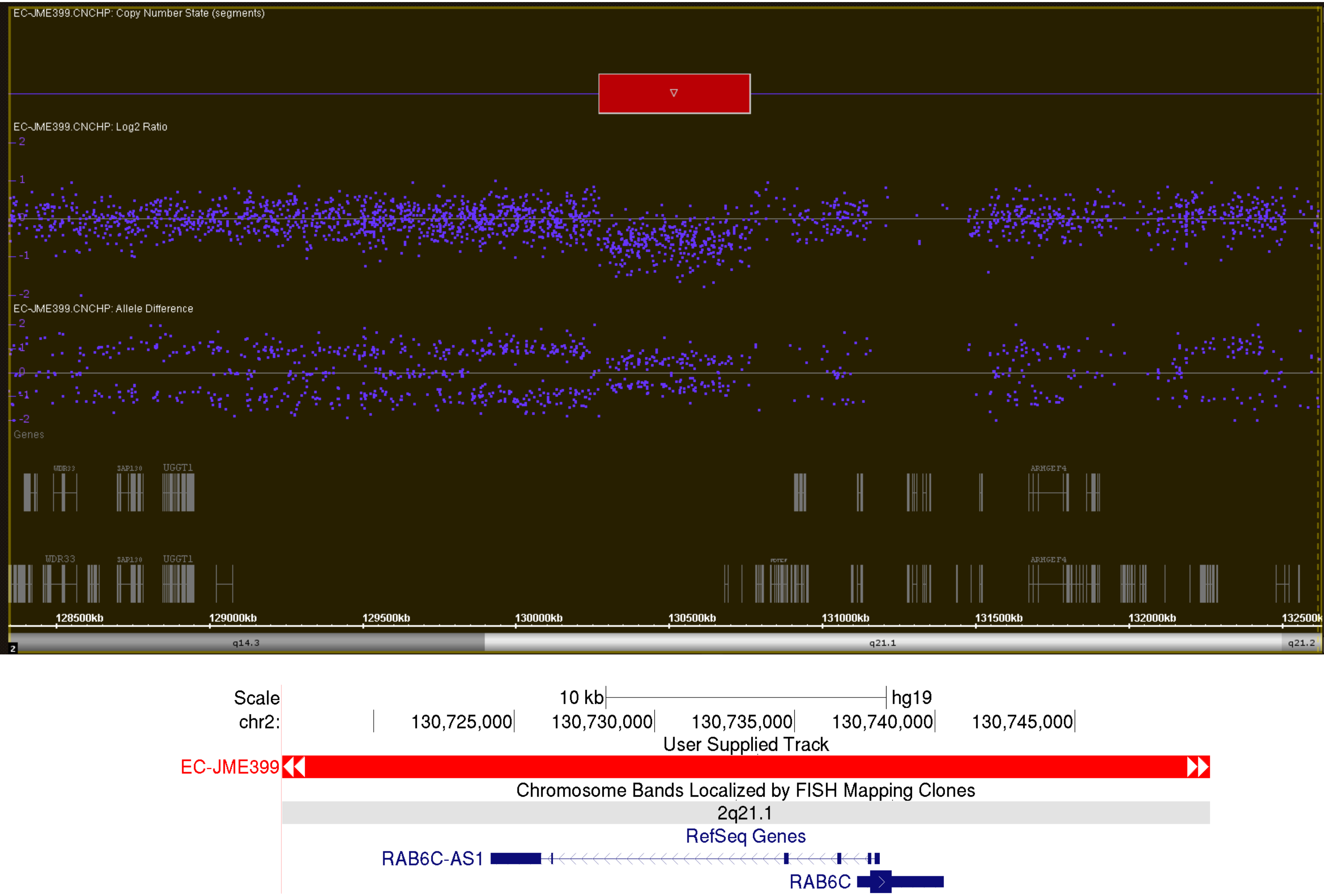

Sample-ID	GGE Syndrome	Chr	Start	End	Candidate gene
EC-JME104	13/JME,gsw	3	85017098	85603757	*CADM2*

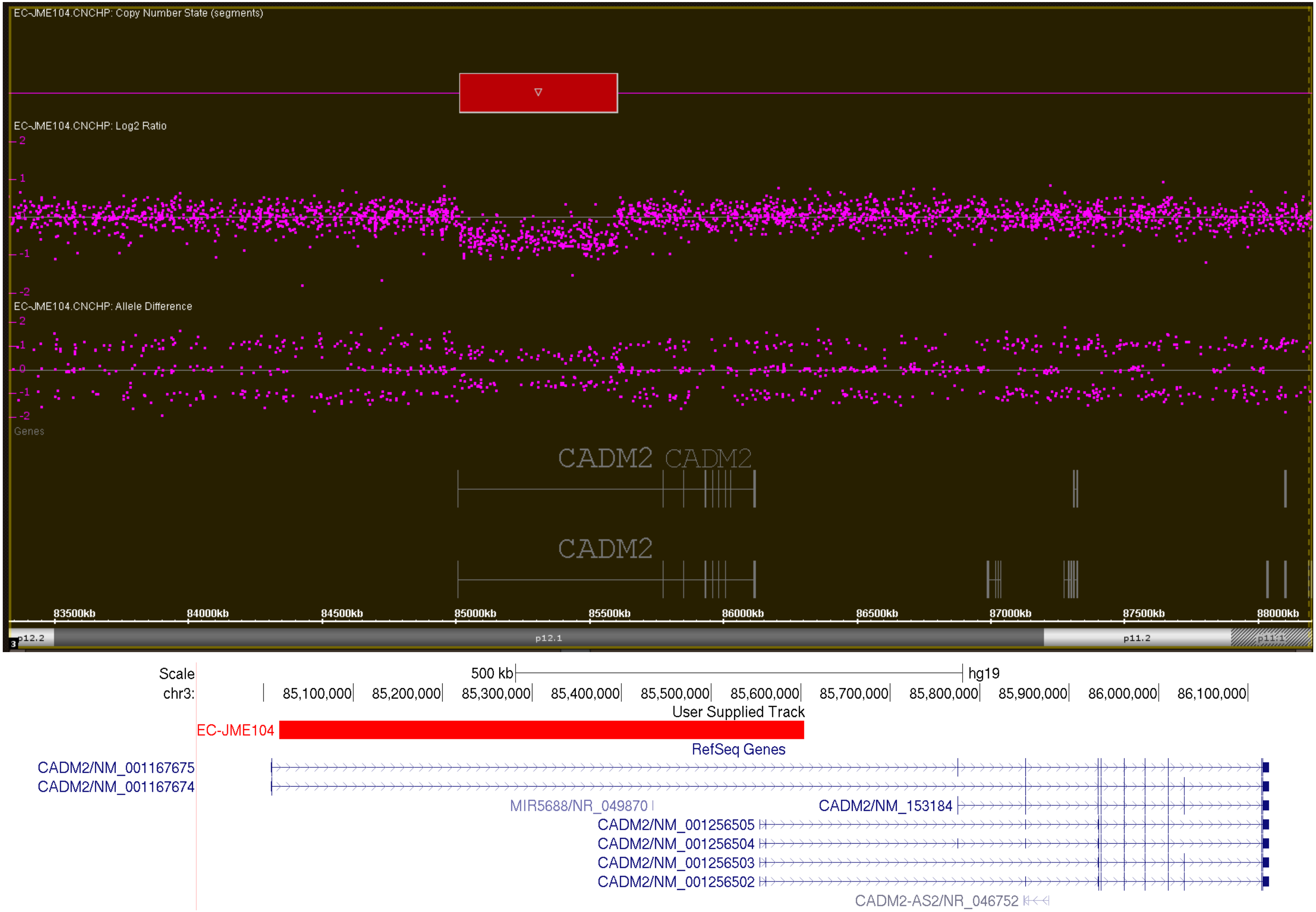

Sample-ID	GGE Syndrome	Chr	Start	End	Candidate gene
EC-CAE040	7/CAE,gsw	3	165317672	166886252	*BCHE*

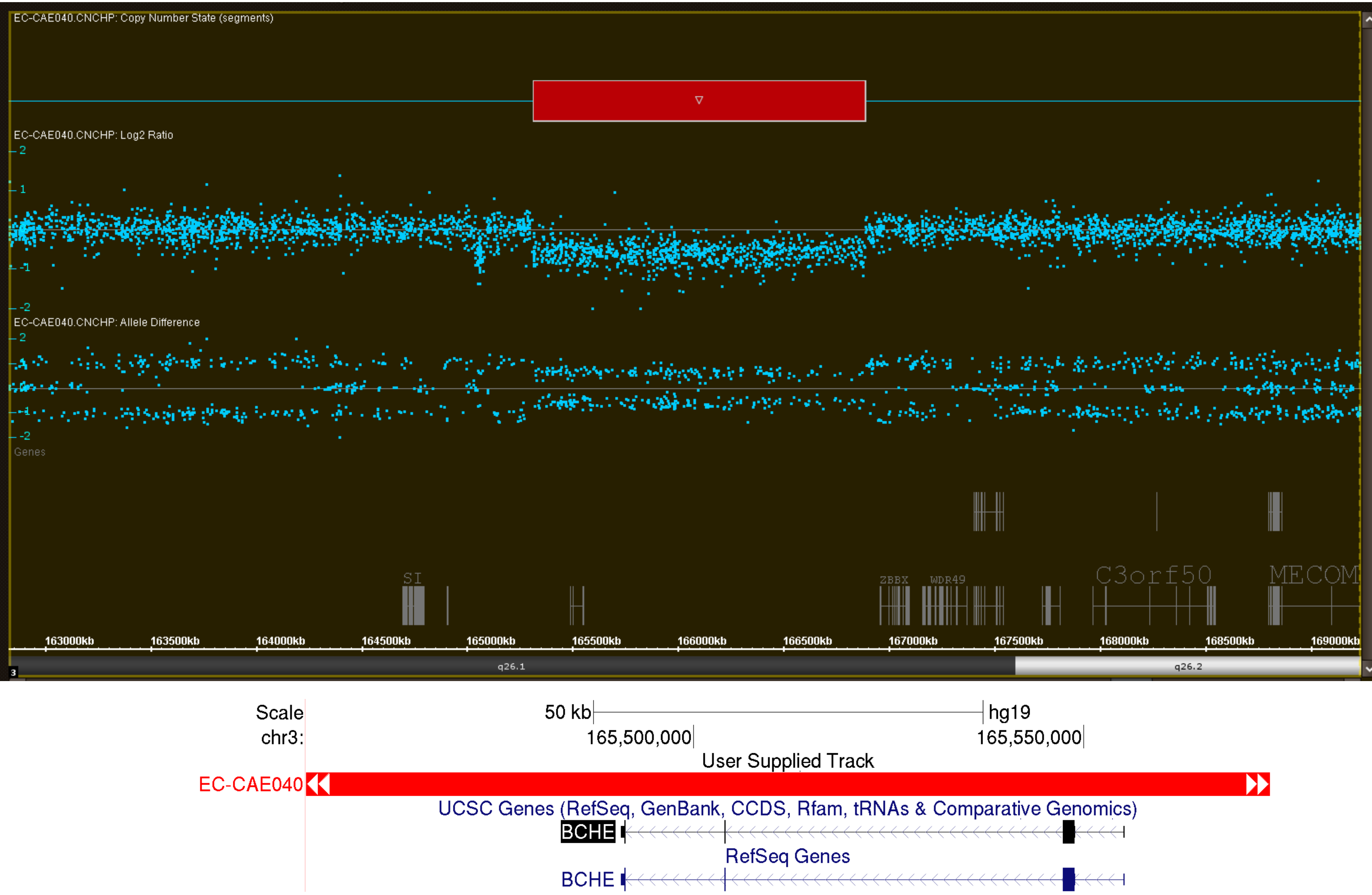

Sample-ID	GGE Syndrome	Chr	Start	End	Candidate gene
EC-JME445	26/JME	4	27778687	31233363	***PCDH7***

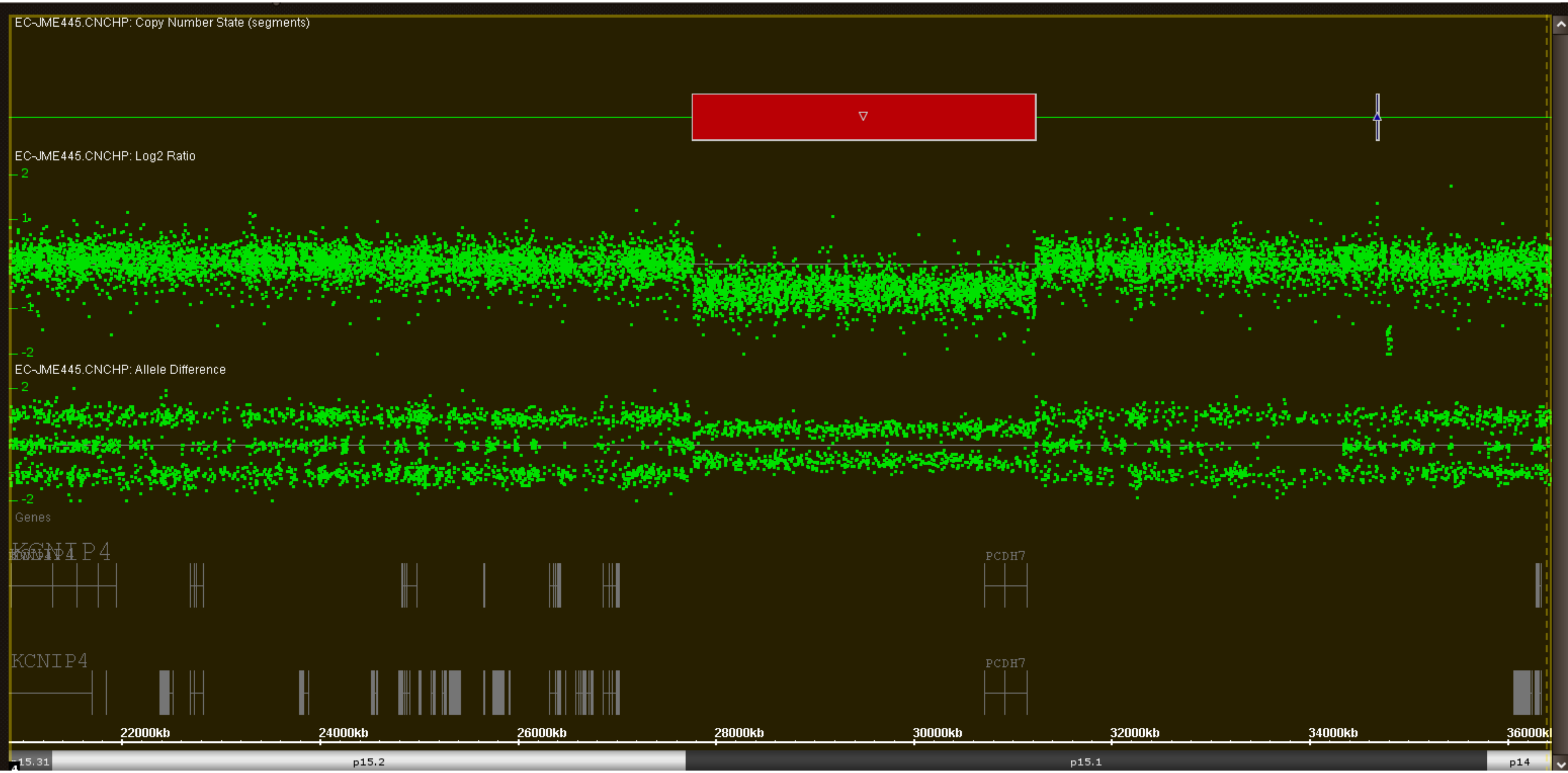

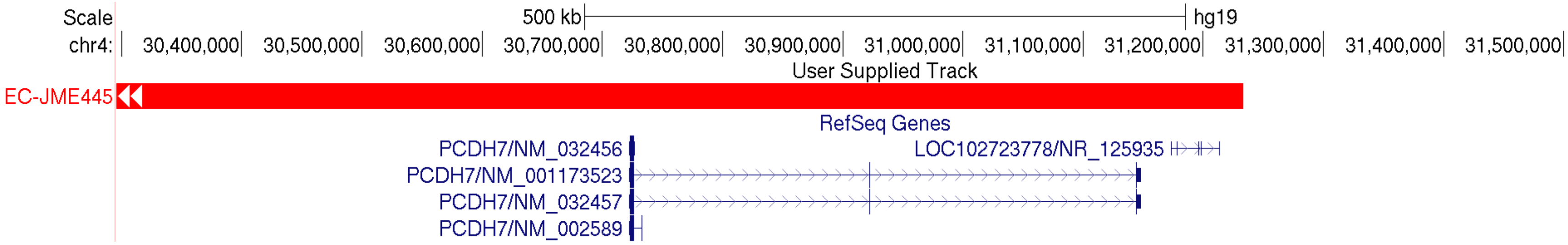

Sample-ID	GGE Syndrome	Chr	Start	End	Candidate gene
EC-CAE099	6/CAE,gsw	5	275875	1257621	*SLC6A19,TERT,BRD9*

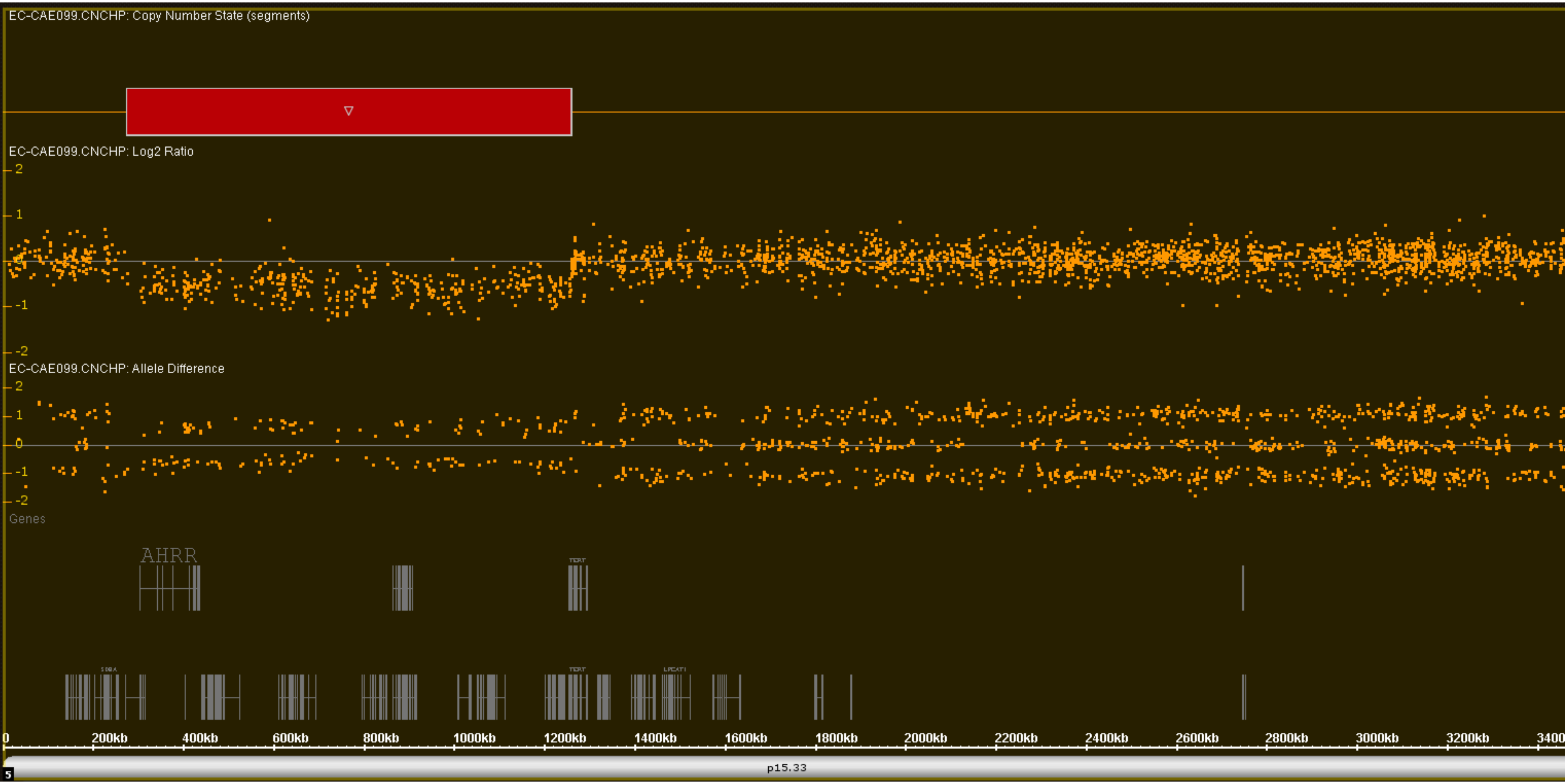

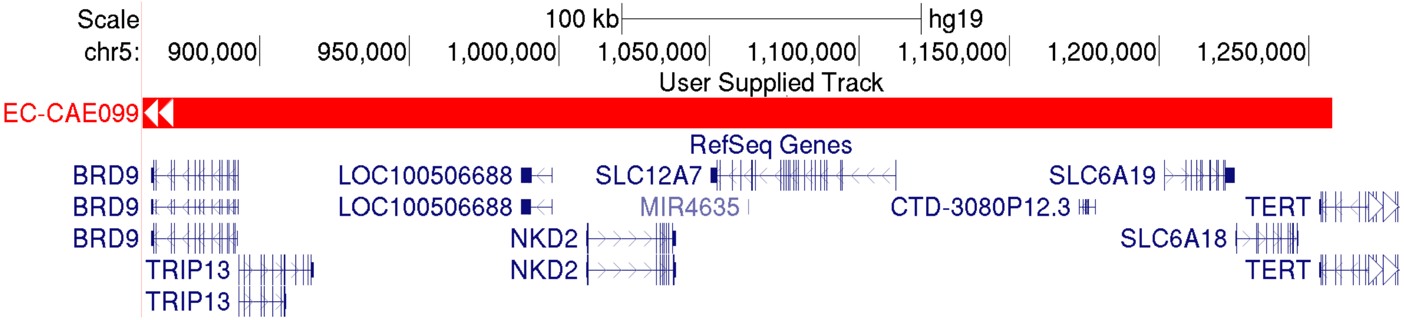

Sample-ID	GGE Syndrome	Chr	Start	End	Candidate gene
EC-JME481	14/JME,gsw	5	28059042	31736582	*DROSHA,CDH6*

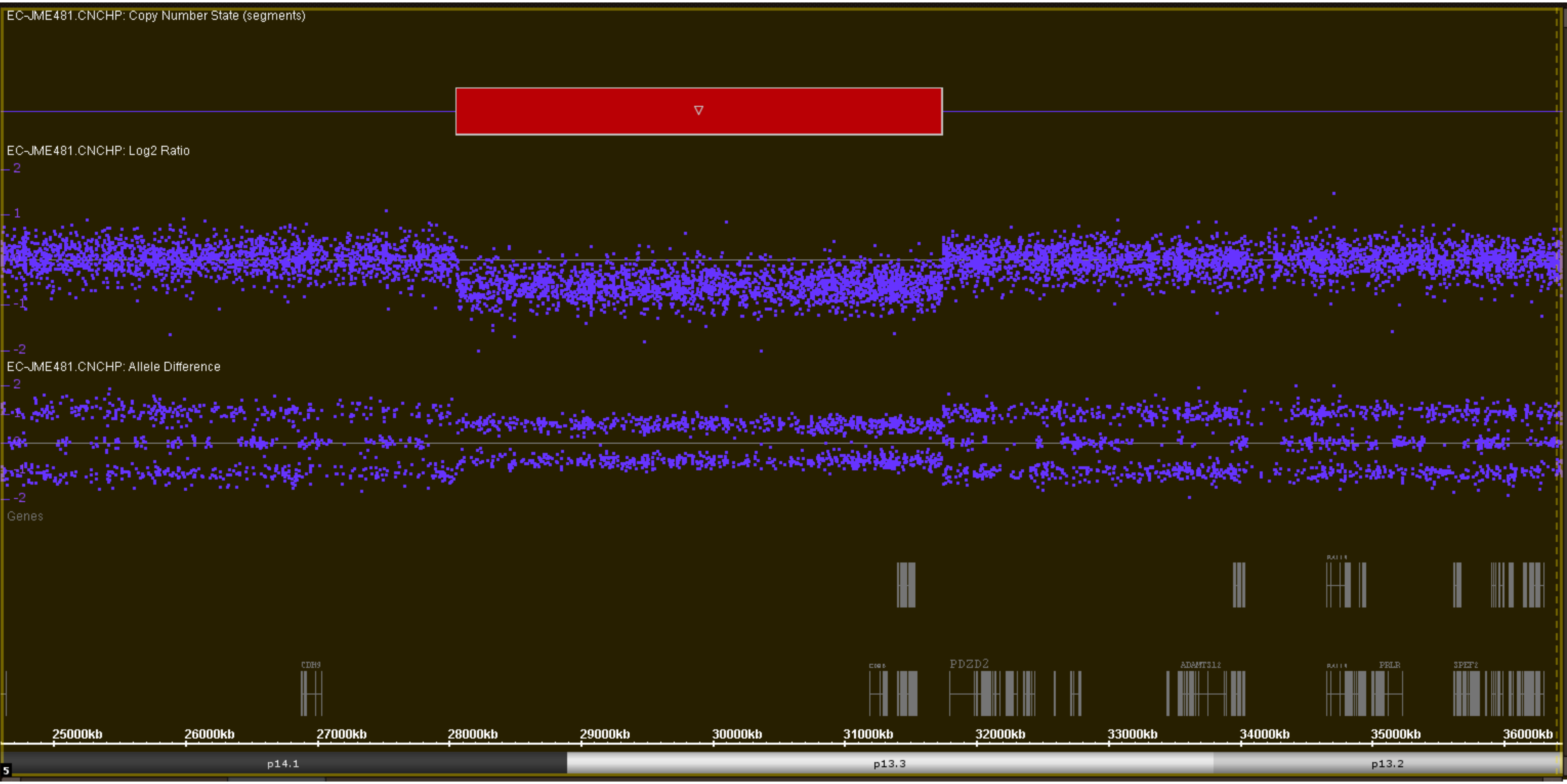

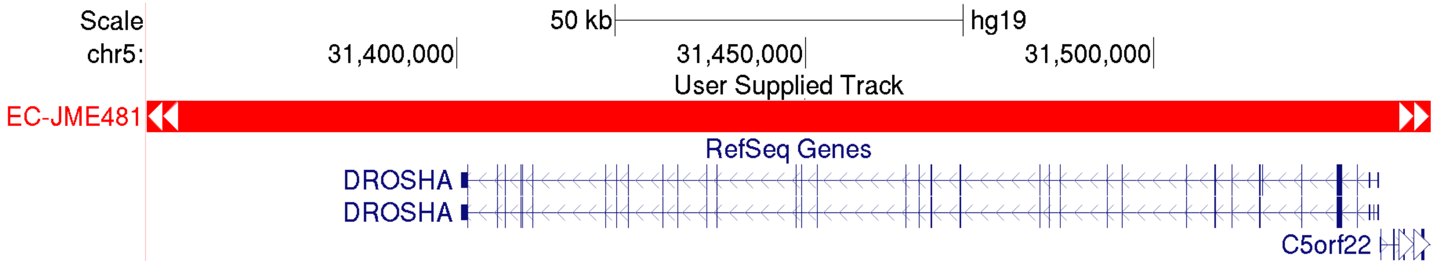

Sample-ID	GGE Syndrome	Chr	Start	End	Candidate gene
EC-CAE347	9/CAE,gsw	6	144444363	146880409	***EPM2A***,*GRM1*

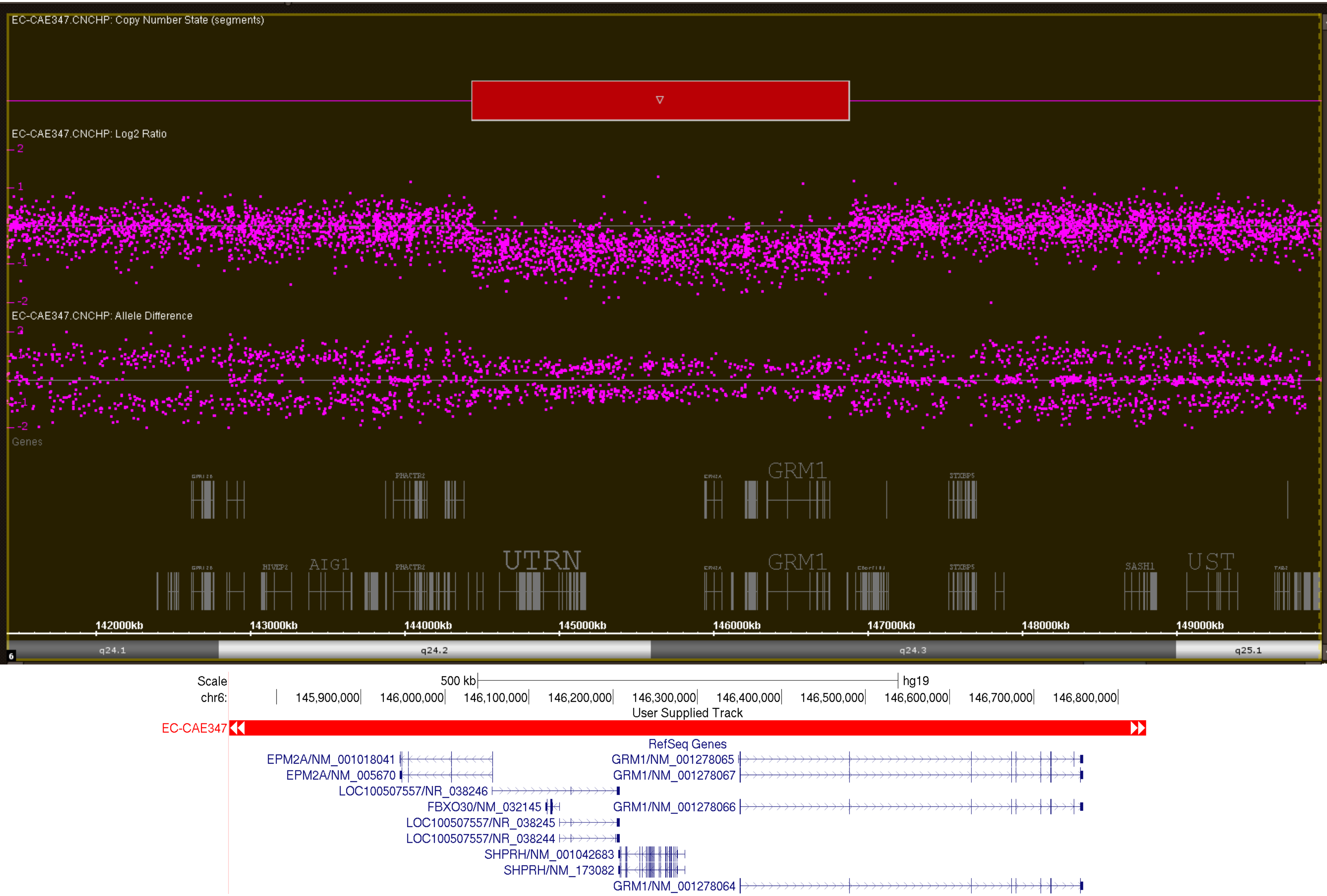

Sample-ID	GGE Syndrome	Chr	Start	End	Candidate gene
EC-CAE204	8/CAE,gsw	6	162801345	163287279	*PARK2*

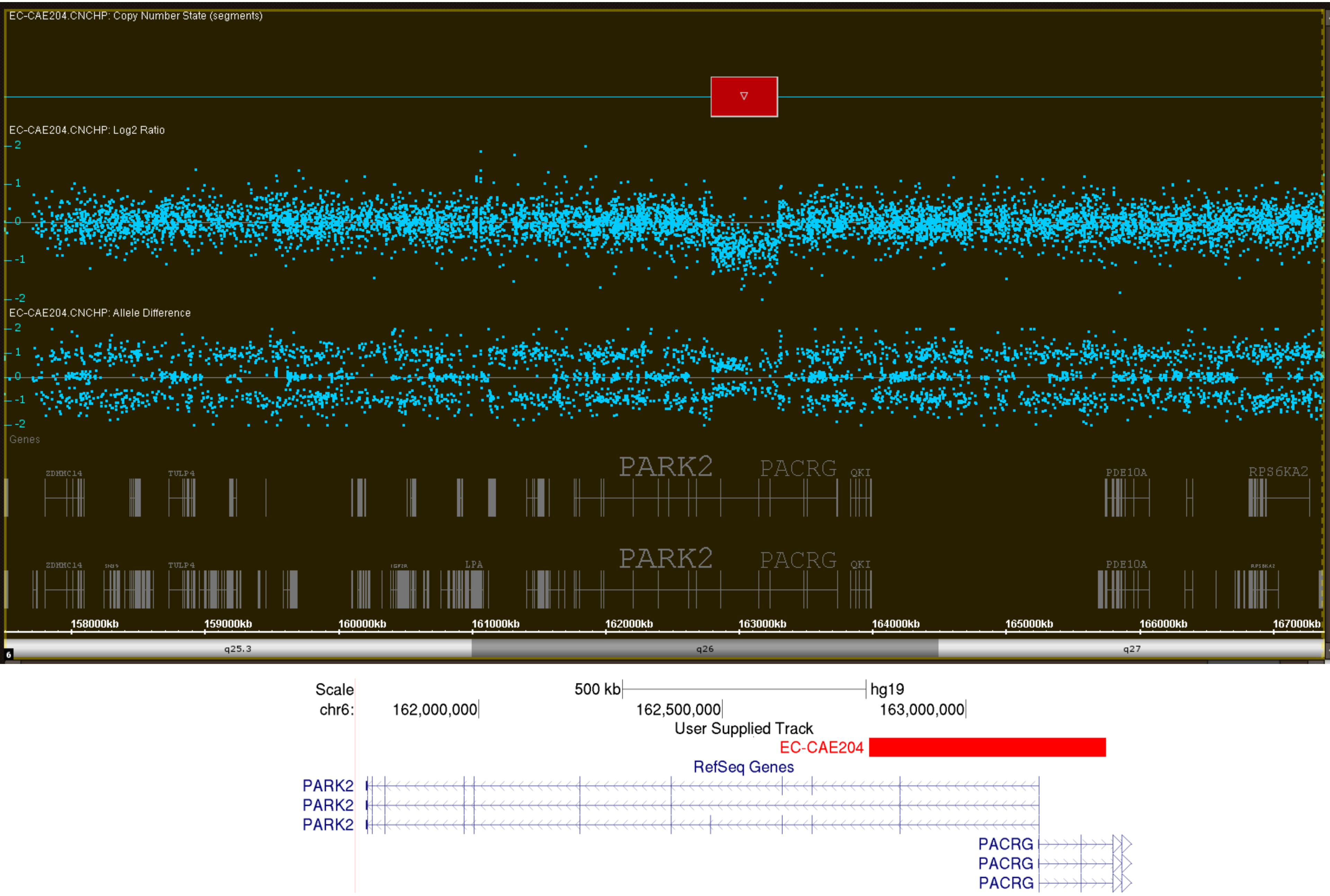

Sample-ID	GGE Syndrome	Chr	Start	End	Candidate gene
EC-JME461	4/CAE	7	124586130	126665734	*GRM8*

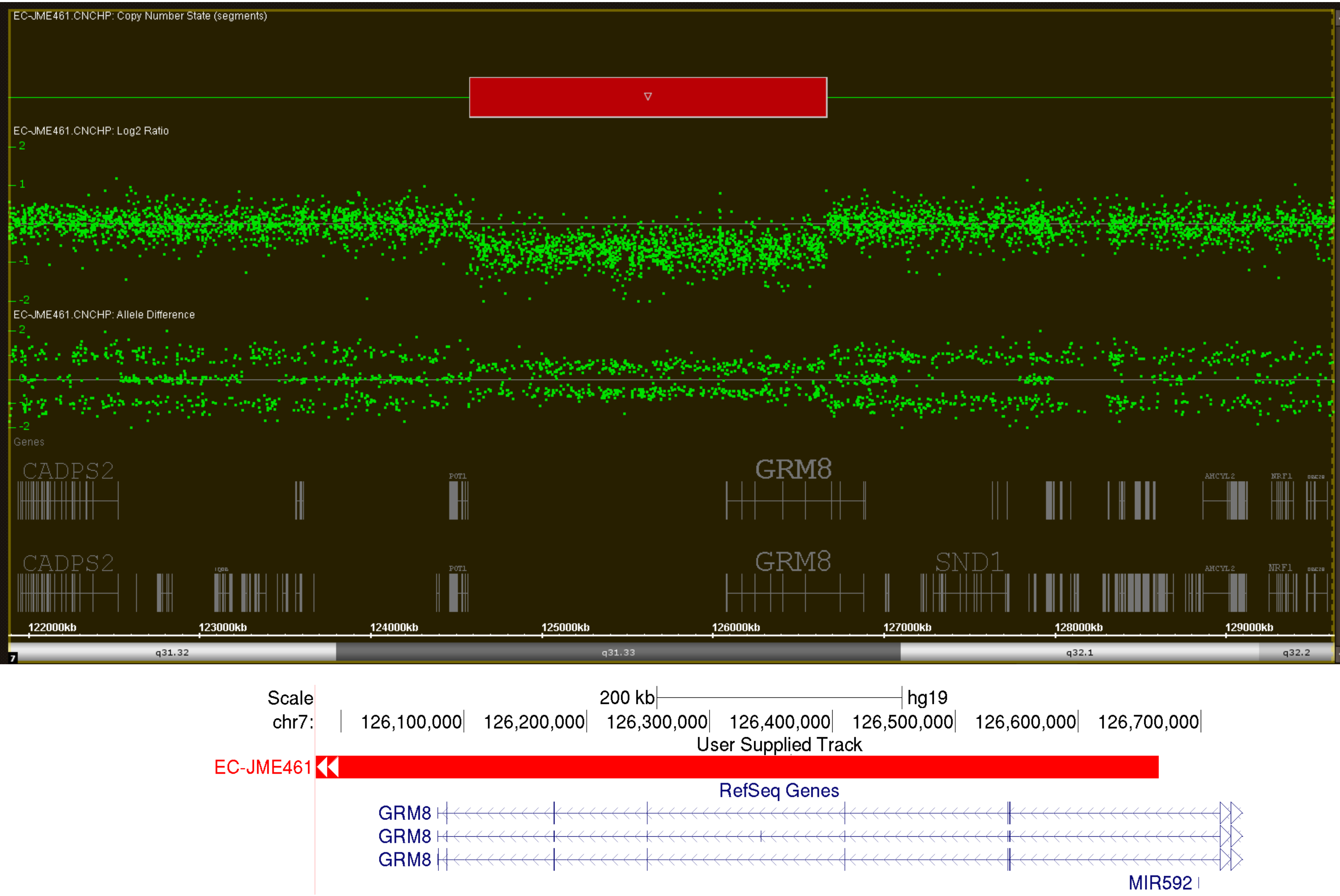

Sample-ID	GGE Syndrome	Chr	Start	End	Candidate gene
EC-CAE158	8/CAE,gsw	7	143223069	143873940	*FAM11C5,FAM115A*

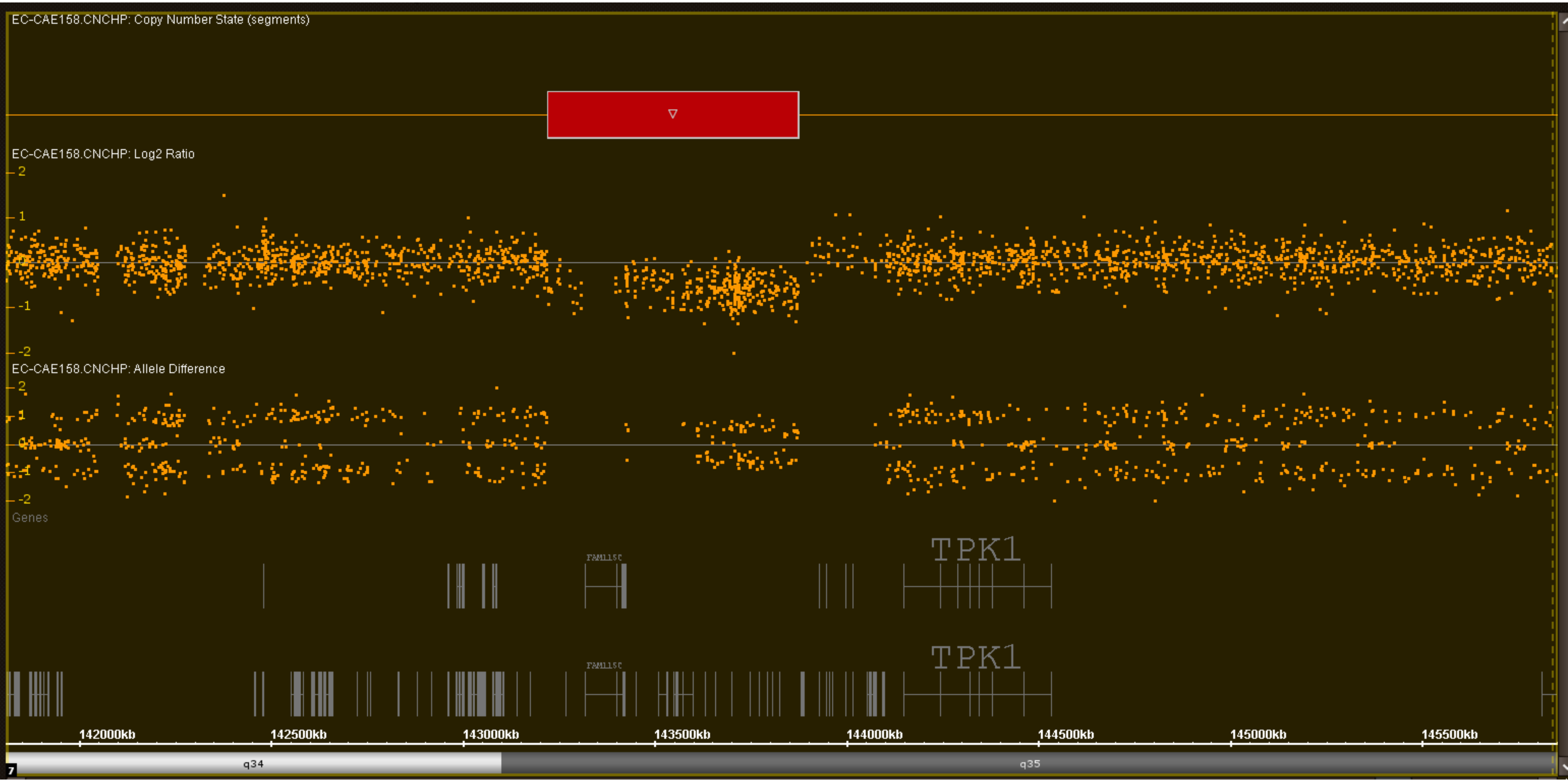

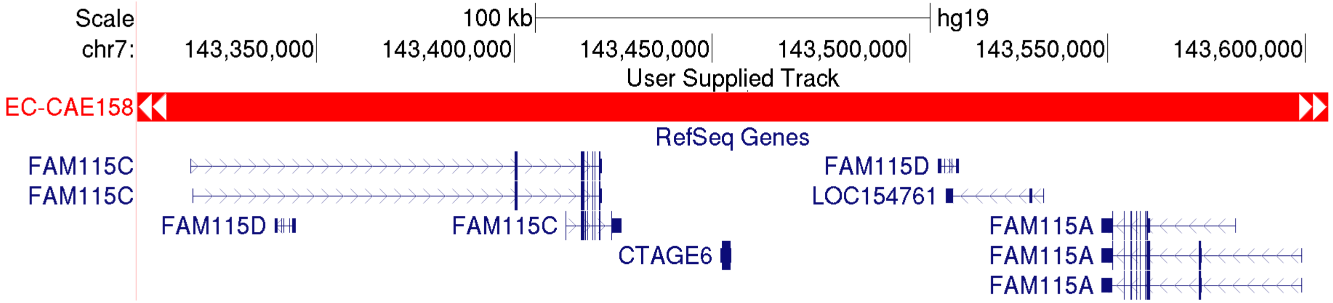

Sample-ID	GGE Syndrome	Chr	Start	End	Candidate gene
EC-EGTCS130	25/EGTCS,gsw	8	99979097	100538070	*VPS13B*

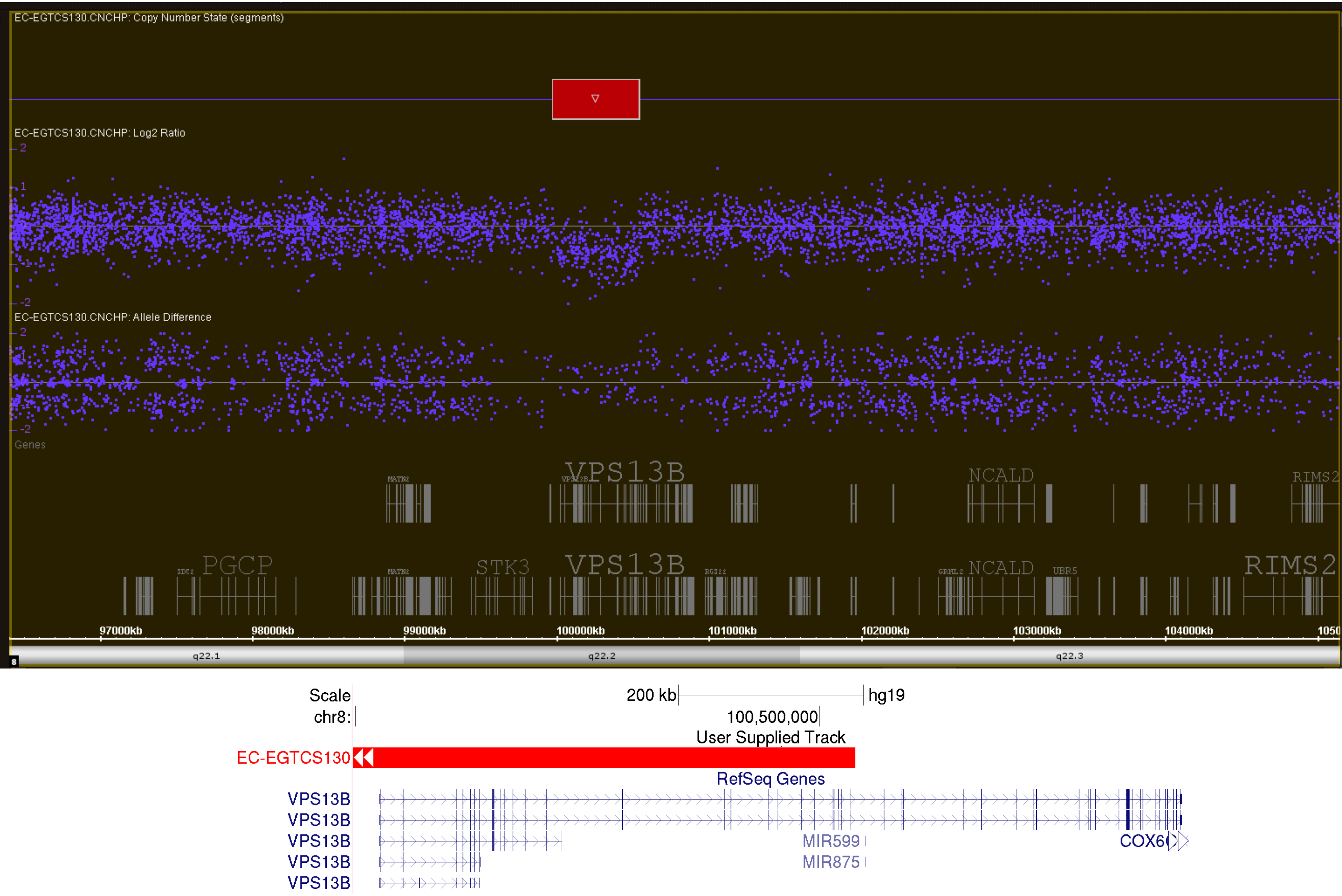

Sample-ID	GGE Syndrome	Chr	Start	End	Candidate gene
EC-JME417	17/JME,gsw	8	142563566	143798641	*TSNARE1,BAI1,ARC*

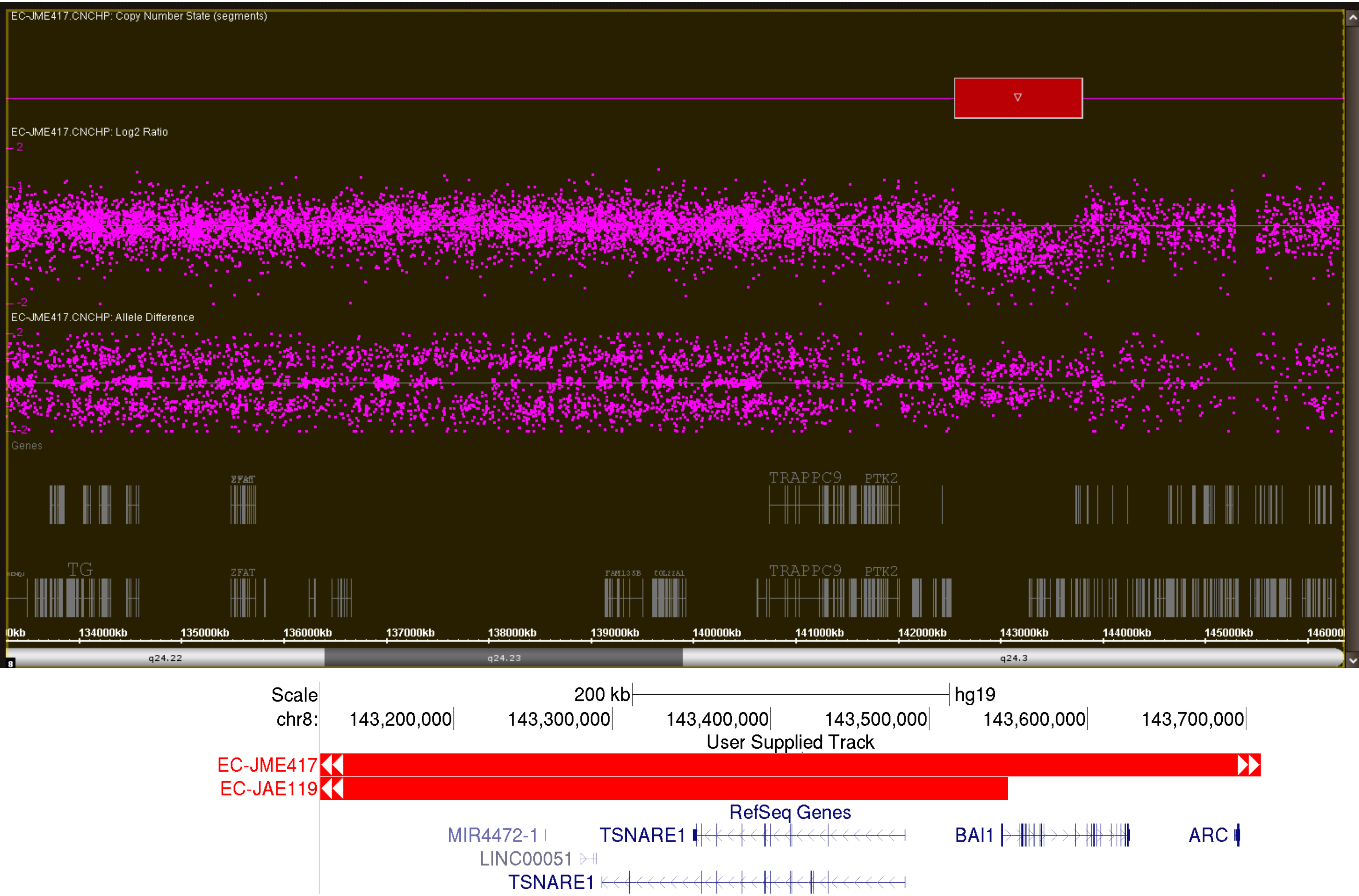

Sample-ID	GGE Syndrome	Chr	Start	End	Candidate gene
EC-JAE119	16/JAE,gsw	8	142850077	143549806	*TSNARE1,BAI1*

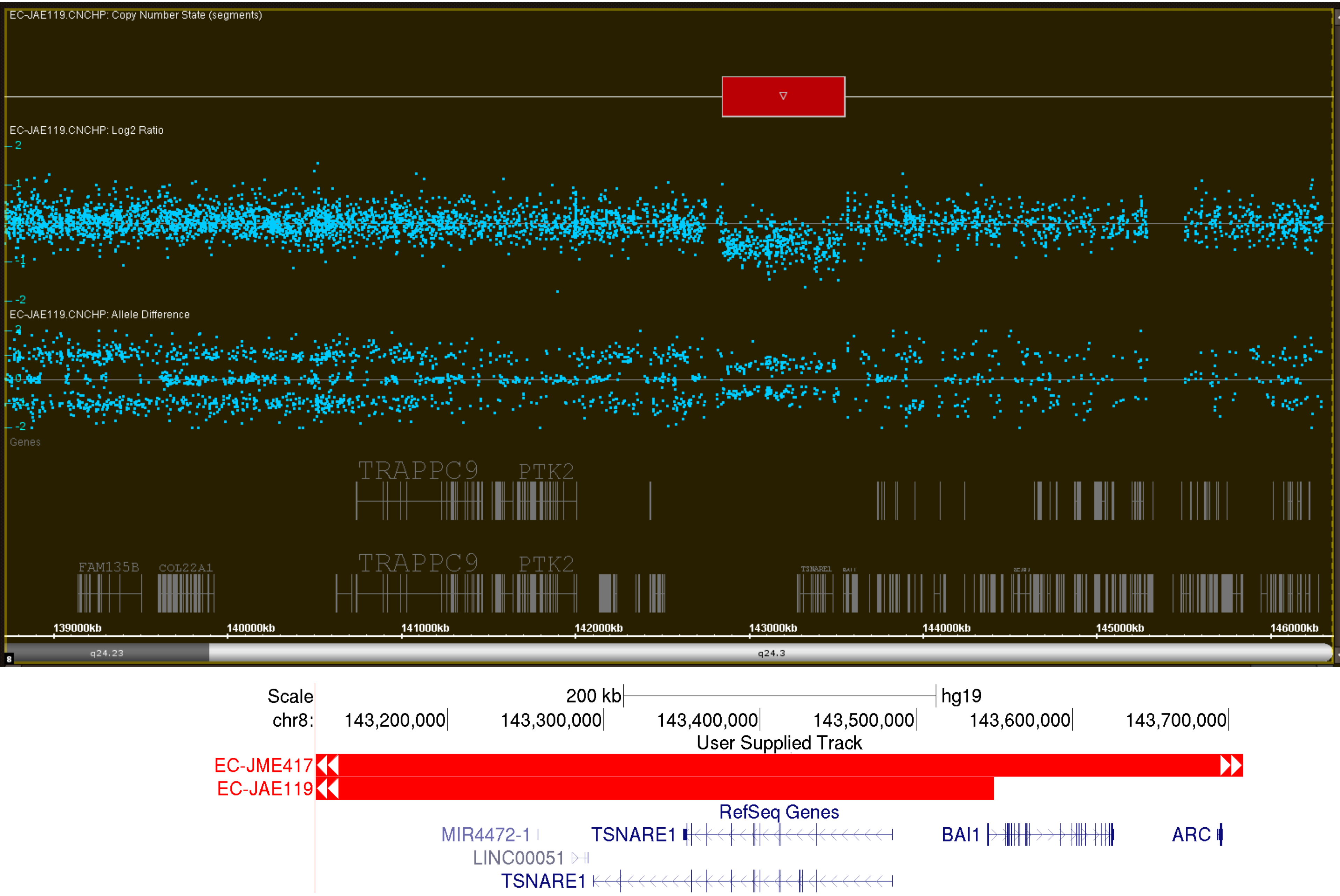

Sample-ID	GGE Syndrome	Chr	Start	End	Candidate gene
EC-CAE300	10/CAE	9	76601085	77182821	***RORB***

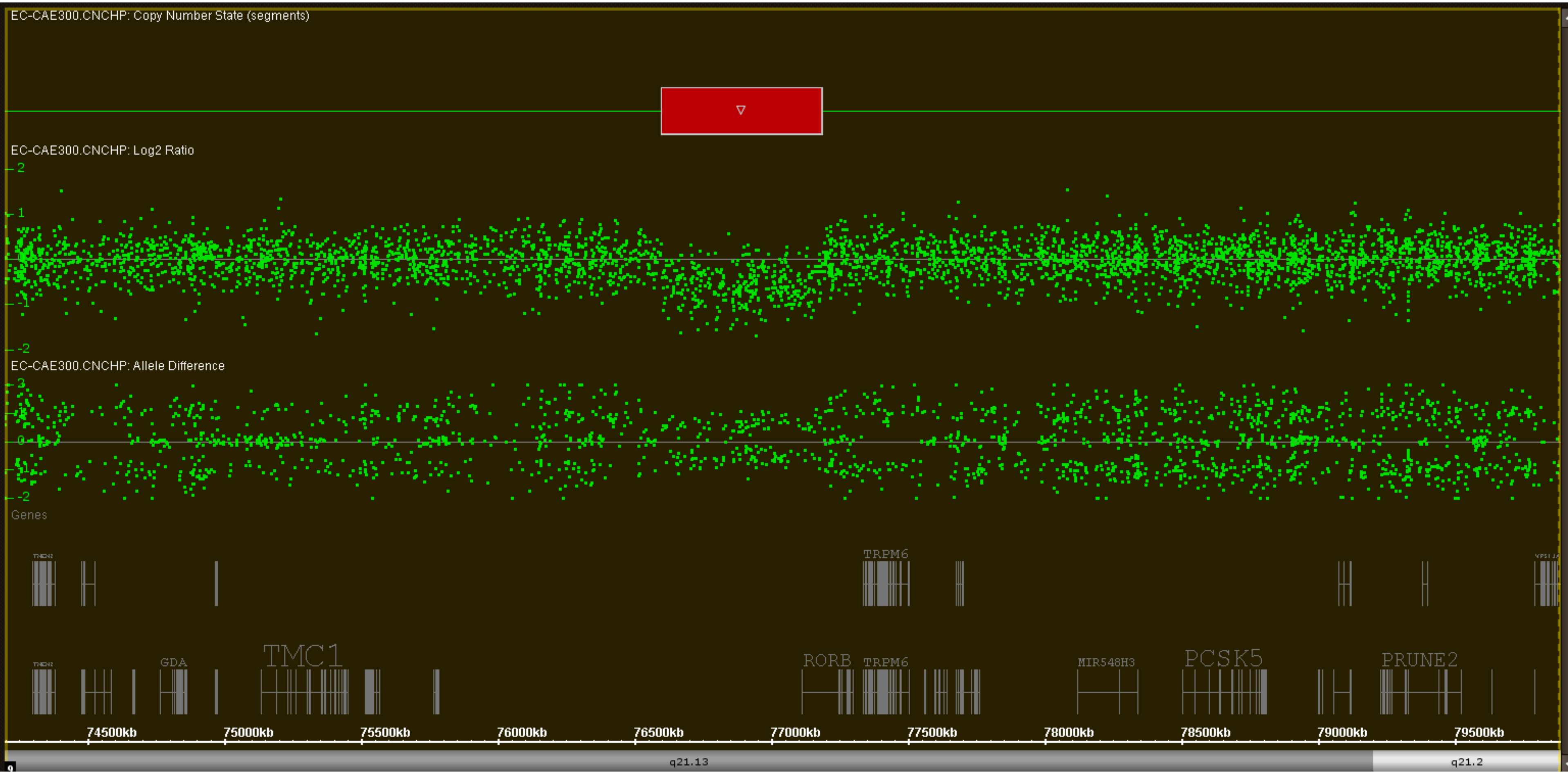

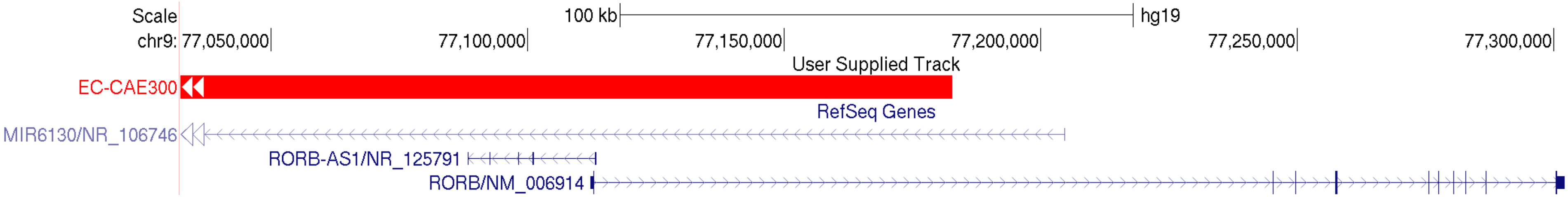

Sample-ID	GGE Syndrome	Chr	Start	End	Candidate gene
EC-JME005	12/JME,gsw	10	27836576	28429513	*MPP7,ARMC4,MKX*

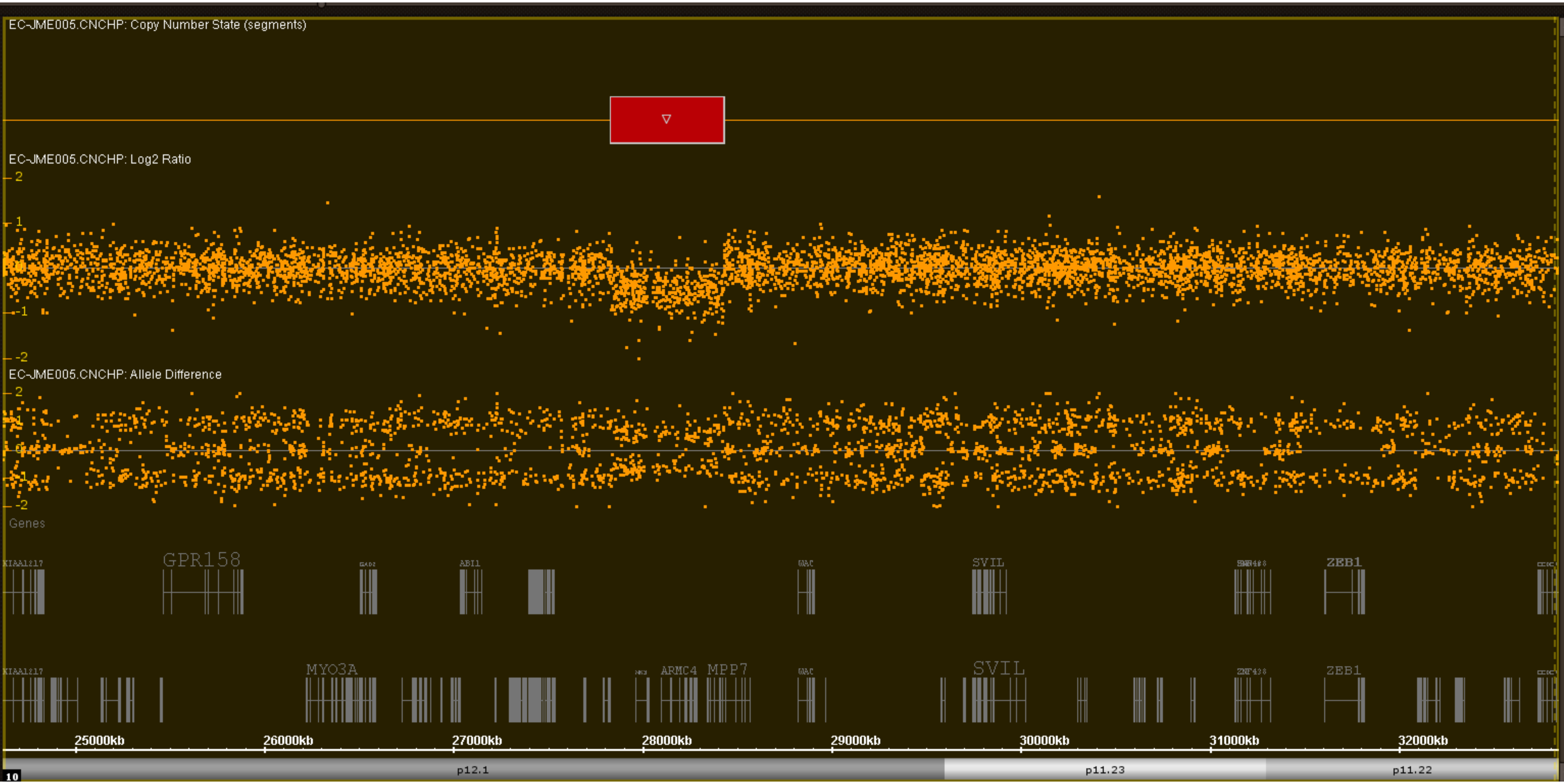

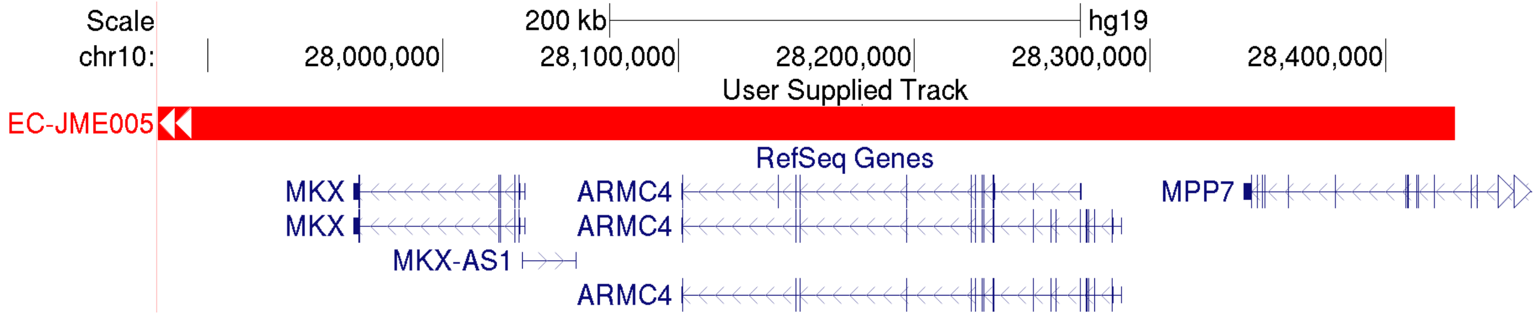

Sample-ID	GGE Syndrome	Chr	Start	End	Candidate gene
EC-JME054	20/JME,gsw	11	4167416	5262622	*C11orf40,TRIM68*

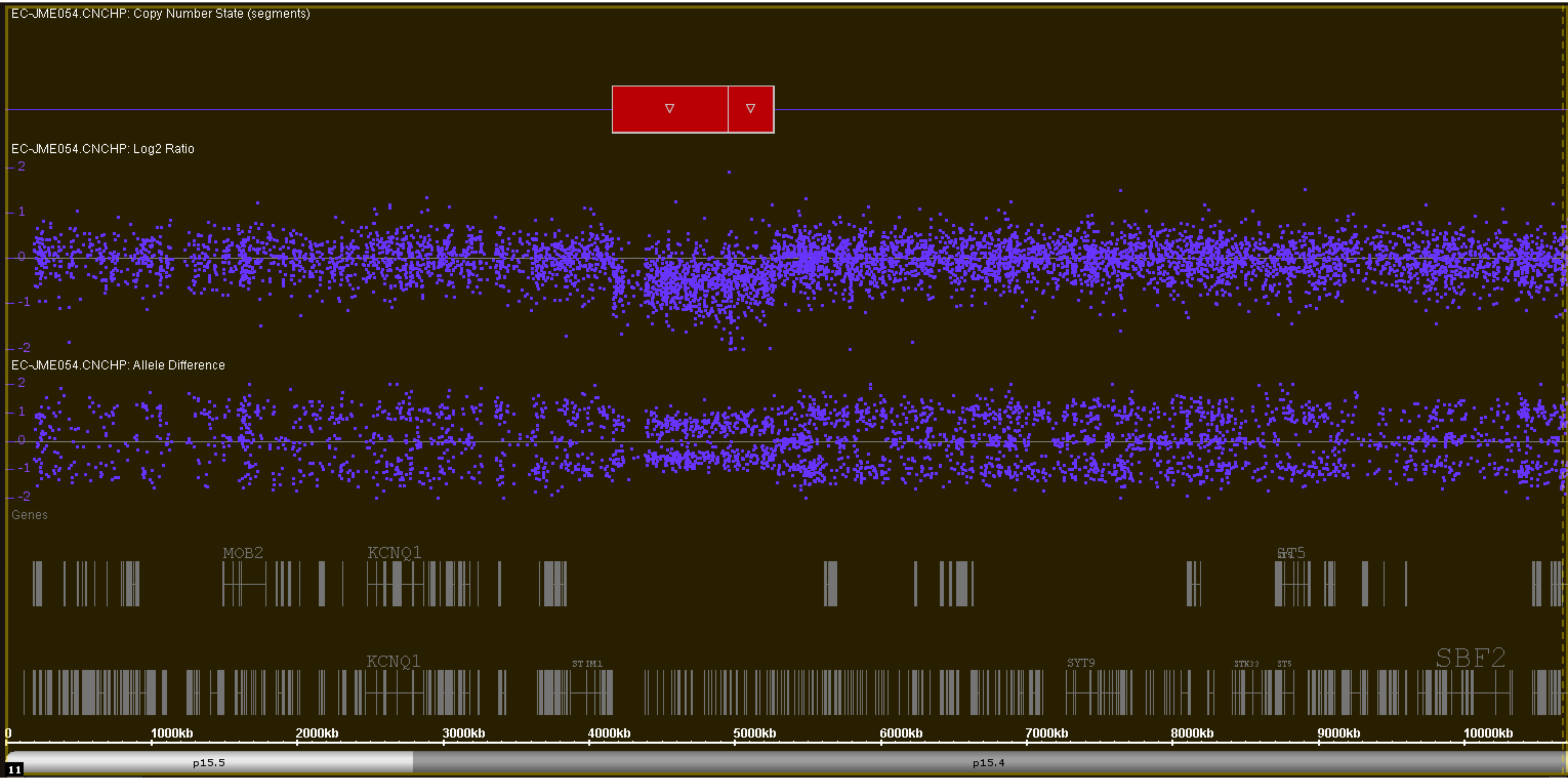

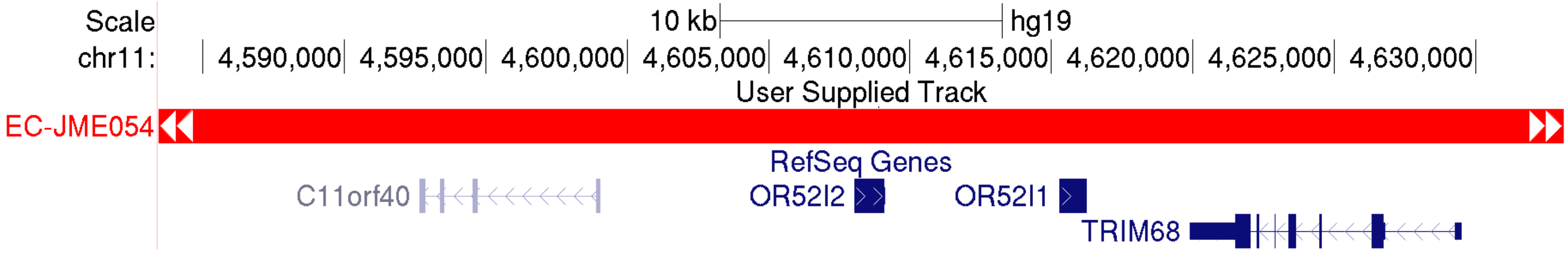

Sample-ID	GGE Syndrome	Chr	Start	End	Candidate gene
EC-JME425	13/JME	12	72135173	73995884	*TBC1D15,TPH2*

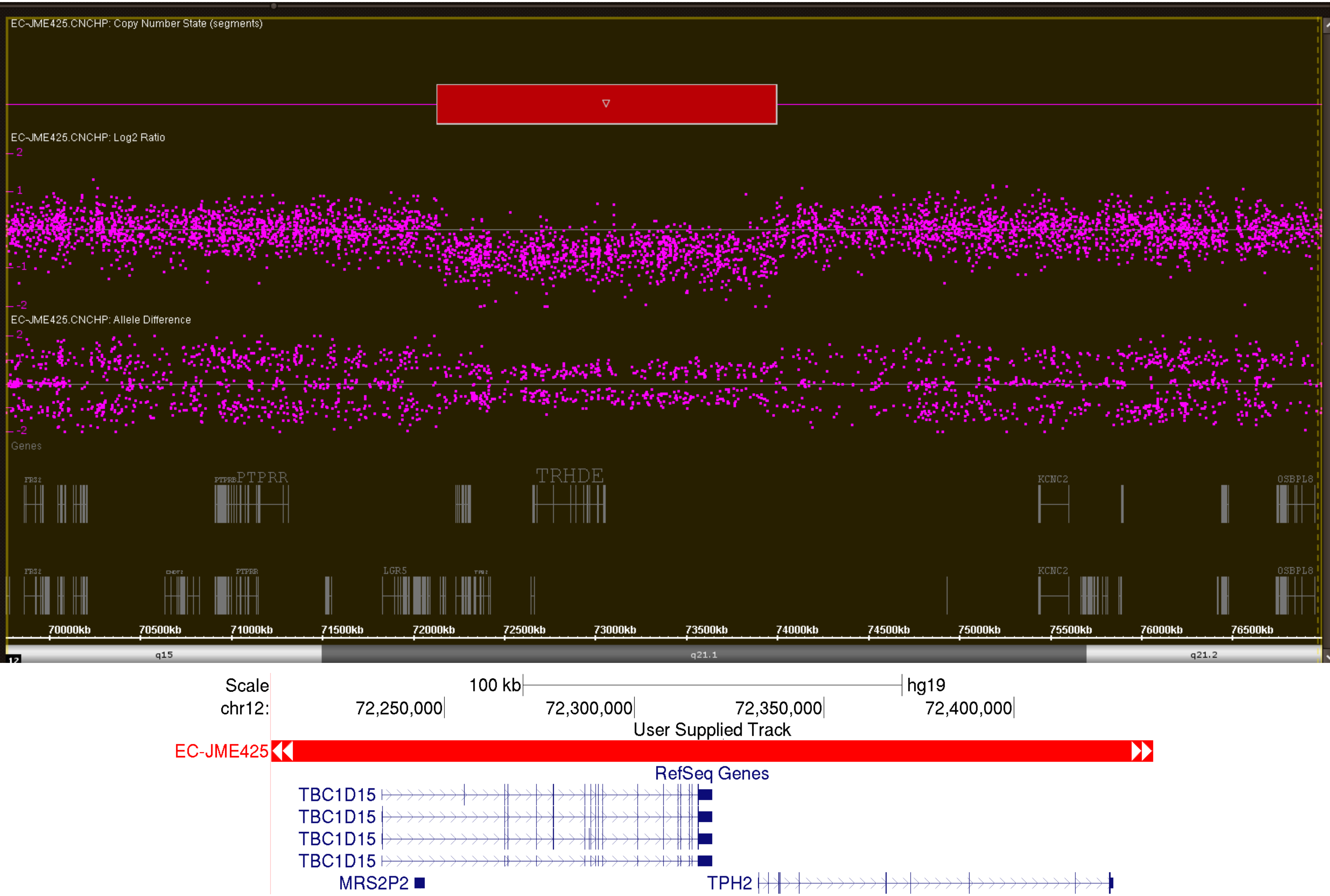

Sample-ID	GGE Syndrome	Chr	Start	End	Candidate gene
EC-JME642	15JME,gsw	15	84915113	85726714	*WDR73,PDE8A*

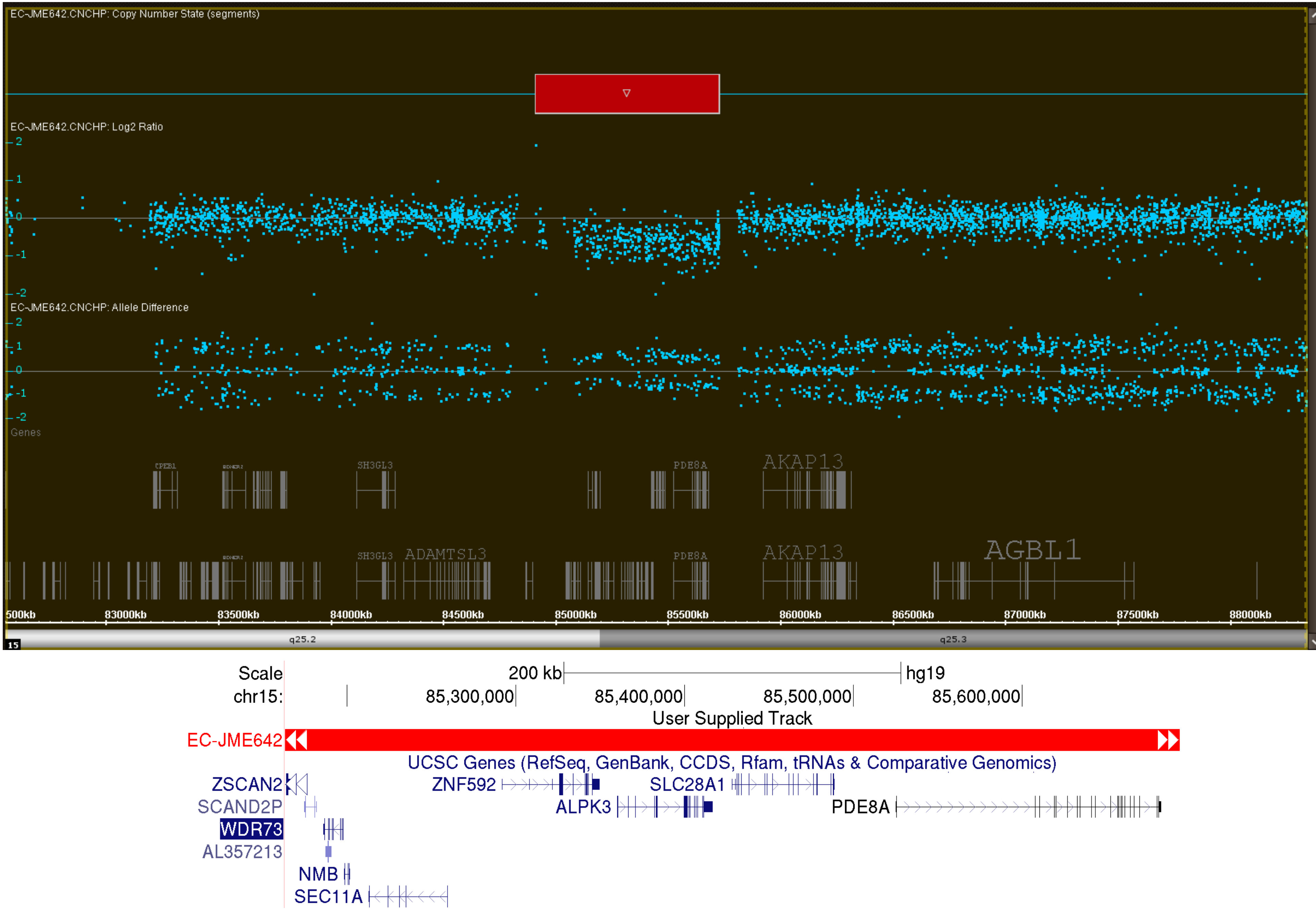

Sample-ID	GGE Syndrome	Chr	Start	End	Candidate gene
EC-CAE286	3/CAE,gsw	16	5615773	6512138	*RBFOX1*

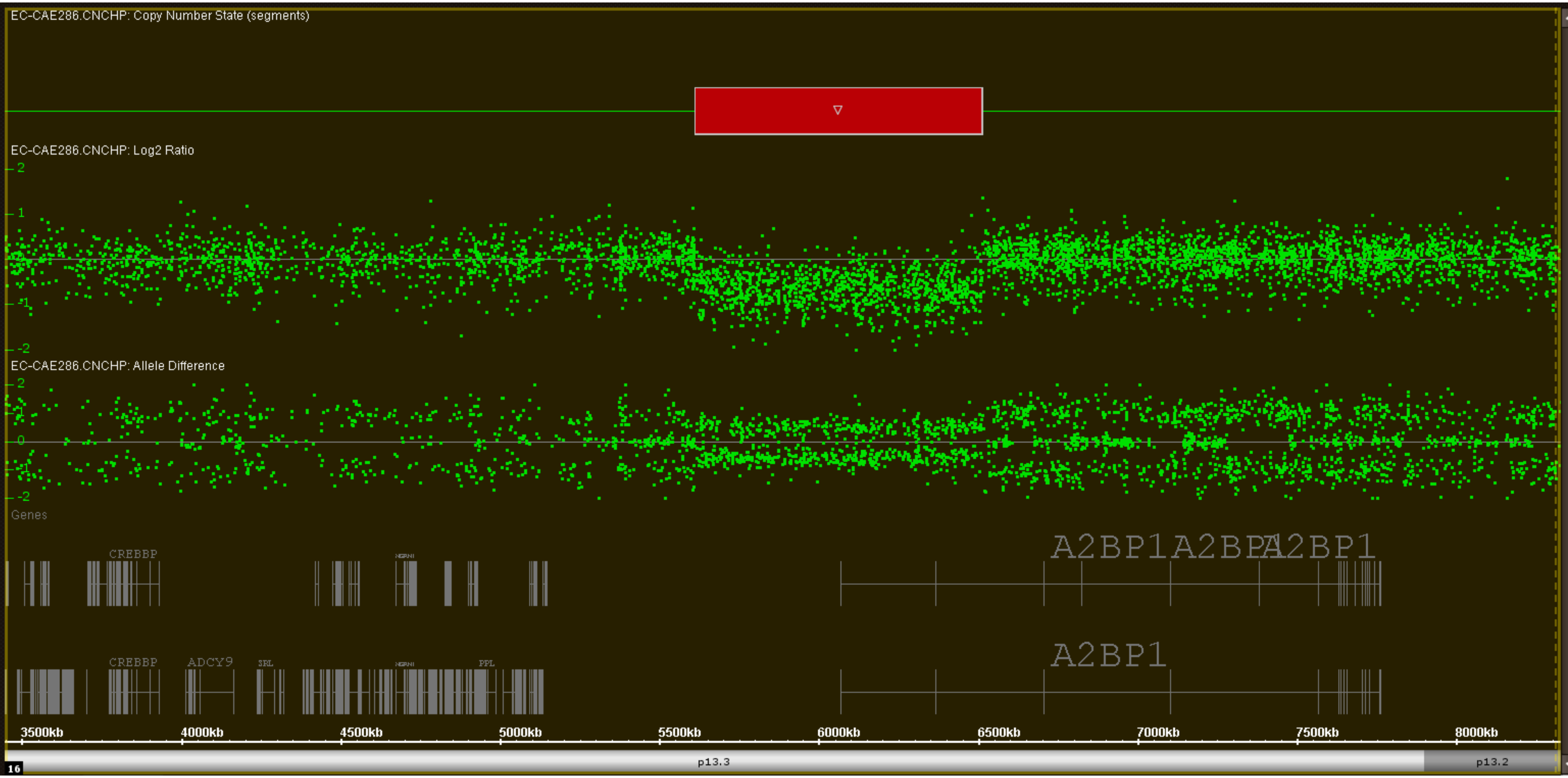

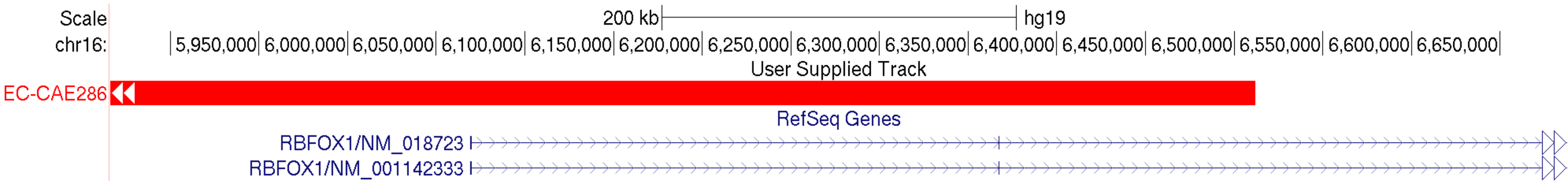

Sample-ID	GGE Syndrome	Chr	Start	End	Candidate gene
EC-CAE226	6/CAE,gsw	18	13982898	14969710	*ZNF519*

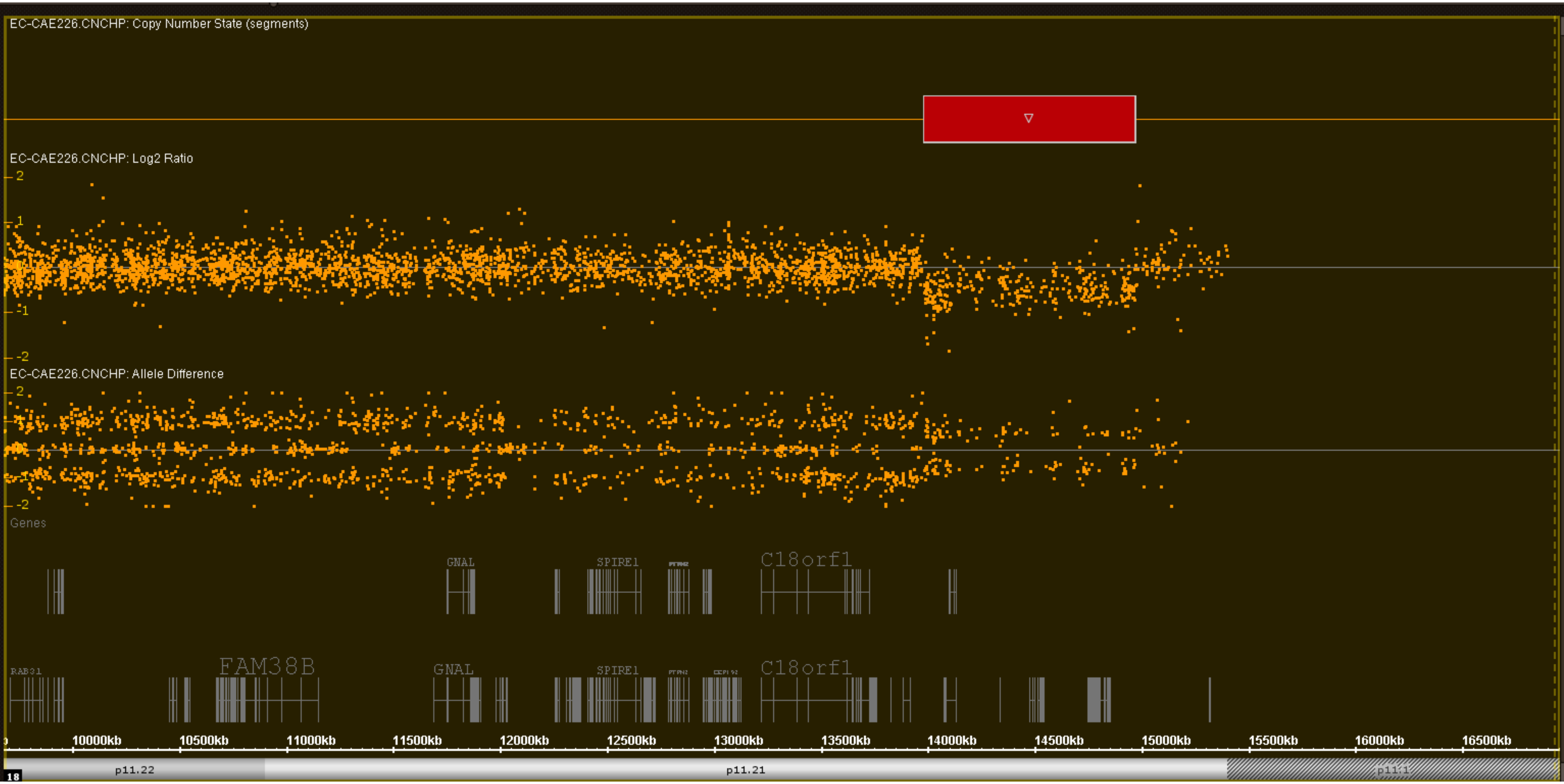

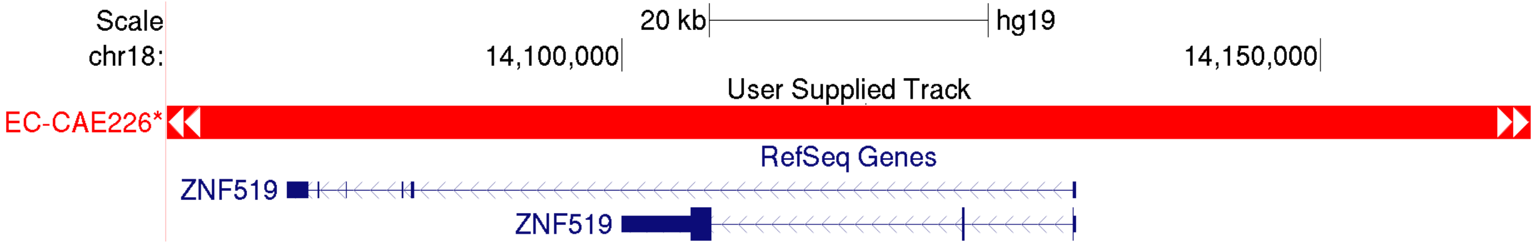

Sample-ID	GGE Syndrome	Chr	Start	End	Candidate gene
EC-CAE161	7/CAE,gsw	20	8099277	8572225	***PLCB1***

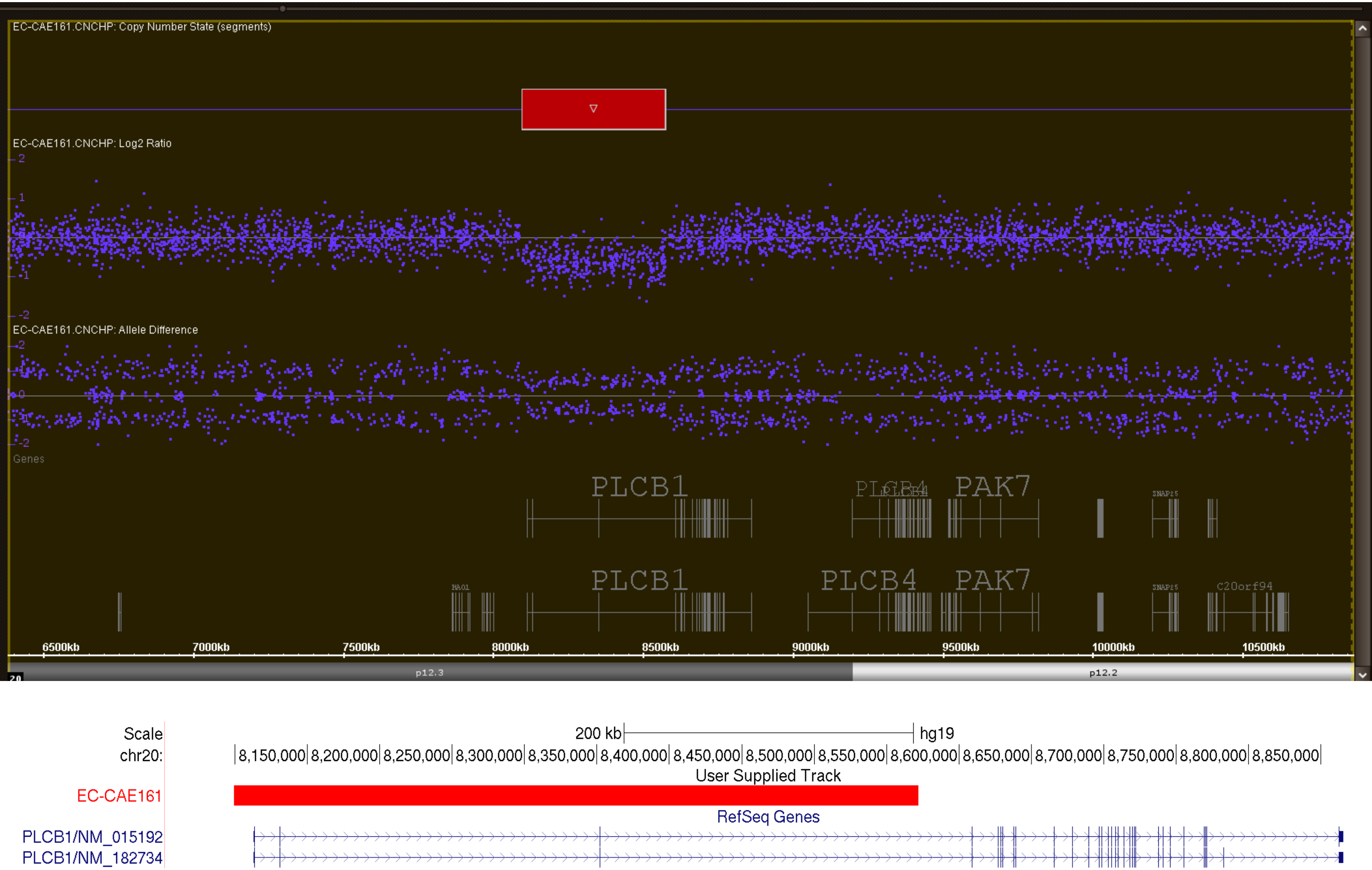

Sample-ID	GGE Syndrome	Chr	Start	End	Candidate gene
EC-EGTCS119	15/EGTCS,gsw	20	14902412	15312347	*MACROD2*

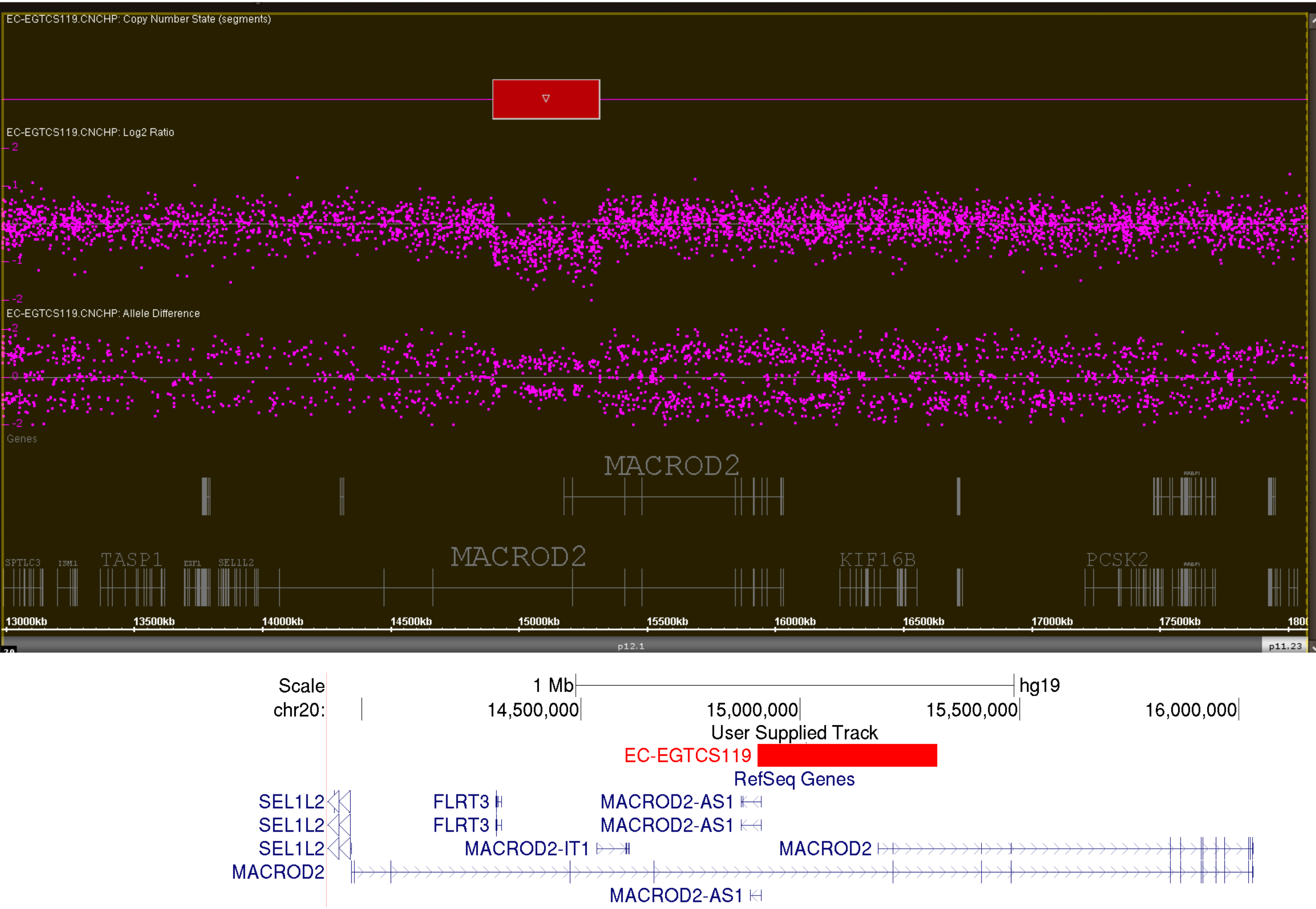

Sample-ID	GGE Syndrome	Chr	Start	End	Candidate gene
EC-JME101	24/JME	21	45866974	48096945	***ADARB1***,*S100B*

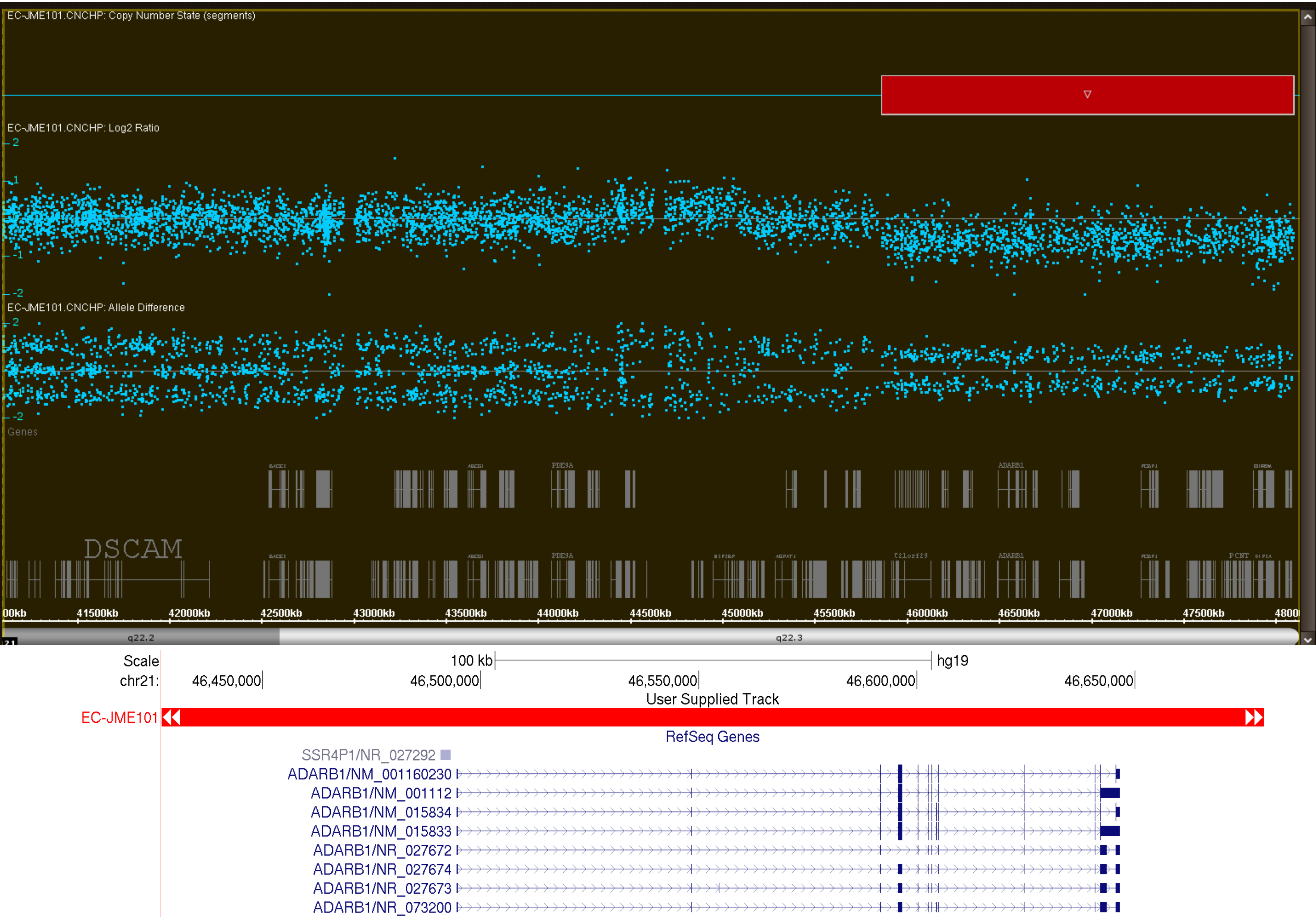

Supplement: S3 Fig — Chr: chromosome, start/end: genomic start and end position of the deleted segment, hg19; GGE, genetic generalised epilepsy; GGE syndromes: CAE: childhood absence epilepsy, JAE: juvenile absence epilepsy, JME: juvenile myoclonic epilepsy, EGMA: epilepsy with generalised tonic-clonic seizures alone predominantly on awakening, EGTCS: epilepsy with generalised tonic-clonic seizures alone, gsw: generalised spike and wave discharges on the electroencephalogram, number/: age-at-onset of afebrile generalised seizures. Bold gene symbols indicate genes previously implicated in epileptogenesis. Signal intensity plots of microdeletions were visualized using the Affymetrix Chromosomal Analyze Suite software. Top track: Red bars represent the computed area of the observed microdeletions. Second track: Signal intensities (Log2 ratios) of a SNP or CN probe are represented by dots, one dot per probe. A segmental decline of consecutive probe signal intensities indicates a genomic deletion. Third track: Allele difference plot, a dot shift to zero indicates loss of heterozygosity which augments high-confidence deletion calling. Bottom track: Survey of the genomic organisation of the microdeletion generated with the University of California, Santa Cruz (UCSC) Genome Browser (http://www.genome.ucsc.edu). Genomic organisation of the microdeletion region: Red bars represent microdeletion size and genomic location in reference to genes affected by the microdeletion (generated with the UCSC Genome Browser, http://www.genome.ucsc.edu). (PDF) [file pgen.1005226.s004.pdf]
