# Supplementary material for: Burden Analysis of Rare Microdeletions Suggests a Strong Impact of Neurodevelopmental Genes in Genetic Generalised Epilepsies
Source: PLoS Genet. 2015 May 7;11(5):e1005226. doi: 10.1371/journal.pgen.1005226 (PMC4423931; doi:10.1371/journal.pgen.1005226)

S4 Fig. Protein-protein interaction networks (PPI) analysis of genes affected by large and rare microdeletions in patients with genetic generalised epilepsies

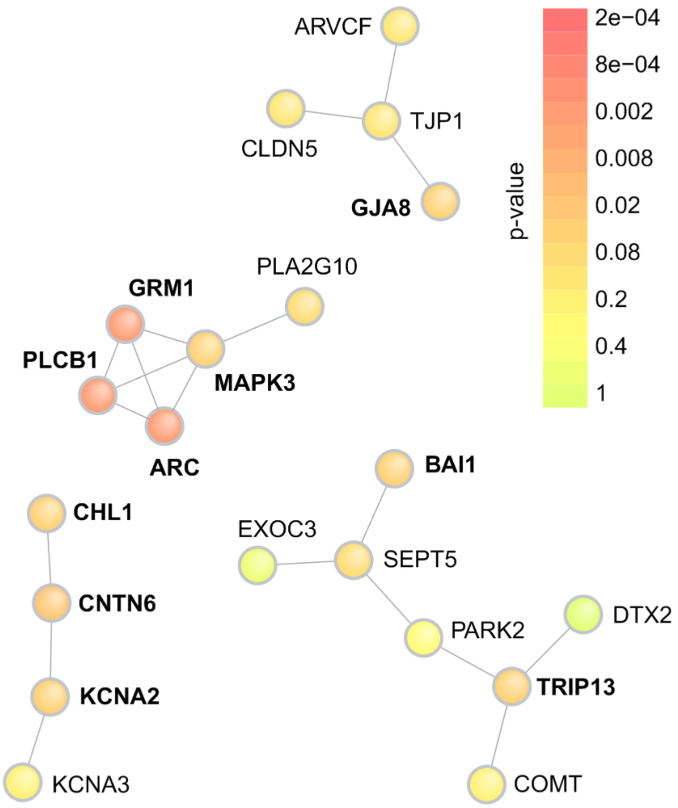

Supplement: S4 Fig — DAPPLE direct networks derived from genes deleted in GGE patients. Depicted are the most connected networks in GGE. Connectivity is coloured from yellow to red describing low to high connection evidence respectively. Significant interactors are marked bold. (PDF) [file pgen.1005226.s005.pdf]
